# Optimizing FAPI homodimers for theranostic applications: The impact of the linker design in DOTAGA.Glu_2_.(FAPI)_2_ and DOTAGA.PEG_2_.Glu.(FAPI)_2_

**DOI:** 10.1007/s00259-026-08078-9

**Published:** 2026-07-15

**Authors:** Naveen Kumar, Adrianna Bilinska, Elena Menéndez, Sarah Stephan, Frank Rösch, Axel Rominger, Eleni Gourni

**Affiliations:** 1 Department of Nuclear Medicine, Inselspital, University Hospital Bern, Bern, Switzerland; 2 Department of Biomedical Research, University of Bern, Bern, Switzerland; 3 Graduate School of Cellular and Biomedical Sciences, University of Bern, Bern, Switzerland; 4 Department of Chemistry–TRIGA site, Johannes Gutenberg-University Mainz, Mainz, Germany

**Keywords:** FAP inhibitor, Homodimeric FAPI, gallium-68, lutetium-177, Fractionated monotherapy, Everolimus, Combination therapy

## Abstract

**Purpose:**

This study evaluated the imaging performance, tumor retention, and therapeutic potential of two new FAPI homodimers, DOTAGA.Glu_2_.(FAPI)_2_ and DOTAGA.PEG_2_.Glu.(FAPI)_2_, specifically designed to improve FAP-targeted tumor retention.

**Methods:**

DOTAGA.Glu_2_.(FAPI)_2_ and DOTAGA.PEG_2_.Glu.(FAPI)_2_ were radiolabeled with gallium-68 and lutetium-177. In vitro studies included lipophilicity, protein binding, saturation binding, internalization and externalization using FAP^+^ CAFs. In vivo evaluation in PC3-mice comprised biodistribution, metabolic stability, blood and organ clearance, PET/SPECT/CT imaging, and autoradiography. Therapeutic efficacy was assessed using fractionated radioligand therapy with [^177^Lu]Lu-DOTAGA.Glu_2_.(FAPI)_2_, as monotherapy or combined with everolimus.

**Results:**

All radioligands showed > 99% radiochemical purity, high FAP affinity (K_d_: 0.9–1.5 nM), hydrophilic profiles (LogD_octanol/PBS_ ≤-3), and low protein binding (< 10%). They exhibited high internalization and low externalization (~ 20% for [^177^Lu]Lu-DOTAGA.Glu_2_.(FAPI)_2_ and ~ 35% for [^177^Lu]Lu-DOTAGA.PEG_2_.Glu.(FAPI)_2_). The ^68^Ga-labeled radiotracers displayed sustained tumor uptake up to 3 h p.i. (> 14%I.A./g) with increasing tumor-to-organ ratios. For the ^177^Lu-labeled analogues the initial uptake was comparable (~ 15%I.A./g at 4 h p.i.), with [^177^Lu]Lu-DOTAGA.Glu_2_.(FAPI)_2_, showing even improved retention compared to [^177^Lu]Lu-DOTAGA.PEG_2_.Glu.(FAPI)_2_ (4.2 ± 0.5 vs. 1.2 ± 0.02%I.A./g at 96 h). Blood clearance was rapid and biphasic, with faster kinetics for DOTAGA.Glu_2_.(FAPI)_2_. For the same compound, tumor half-life was significantly prolonged (44.5 vs. 19 h), resulting in 2-fold higher tumor exposure (AUC: 971 ± 74 vs. 542 ± 28%I.A.×h/g). Imaging confirmed high tumor-to-background ratios, while autoradiography revealed heterogeneous intratumoral FAP distribution. Fractionated therapy significantly inhibited tumor growth and improved survival, further enhanced by everolimus.

**Conclusion:**

DOTAGA.Glu_2_.(FAPI)_2_ demonstrates superior tumor retention, favorable pharmacokinetics, and enhanced therapeutic efficacy, supporting its potential for clinical translation.

**Supplementary Information:**

The online version contains supplementary material available at https://doi.org/10.1007/s00259-026-08078-9.

## Introduction

Fibroblast activation protein (FAP) is a membrane-bound serine protease highly expressed in cancer-associated fibroblasts (CAFs), which are abundant in the stroma of many solid tumors with comparatively lower and more restricted expression in healthy adult tissues [1, 2]. This expression profile, together with its presence across a wide range of tumor types, makes FAP an attractive target for molecular imaging and targeted radionuclide therapy (TRT) with pan-tumor applicability [3, 4]. Radiolabeled FAP inhibitors (FAPIs), particularly monomers, have been widely investigated for positron emission tomography (PET) imaging, showing rapid tumor uptake and high tumor-to-background ratios across various malignancies [5–16]. Several monomeric FAPIs have already been utilized clinically for staging, therapy monitoring, and theranostic applications [5–7, 10, 14, 15, 17]. However, despite these favourable imaging properties, their relatively short tumor retention constrains their therapeutic efficacy when radiolabeled with β-emitting radionuclides such as lutetium-177 [13].

**Fig. 1:**
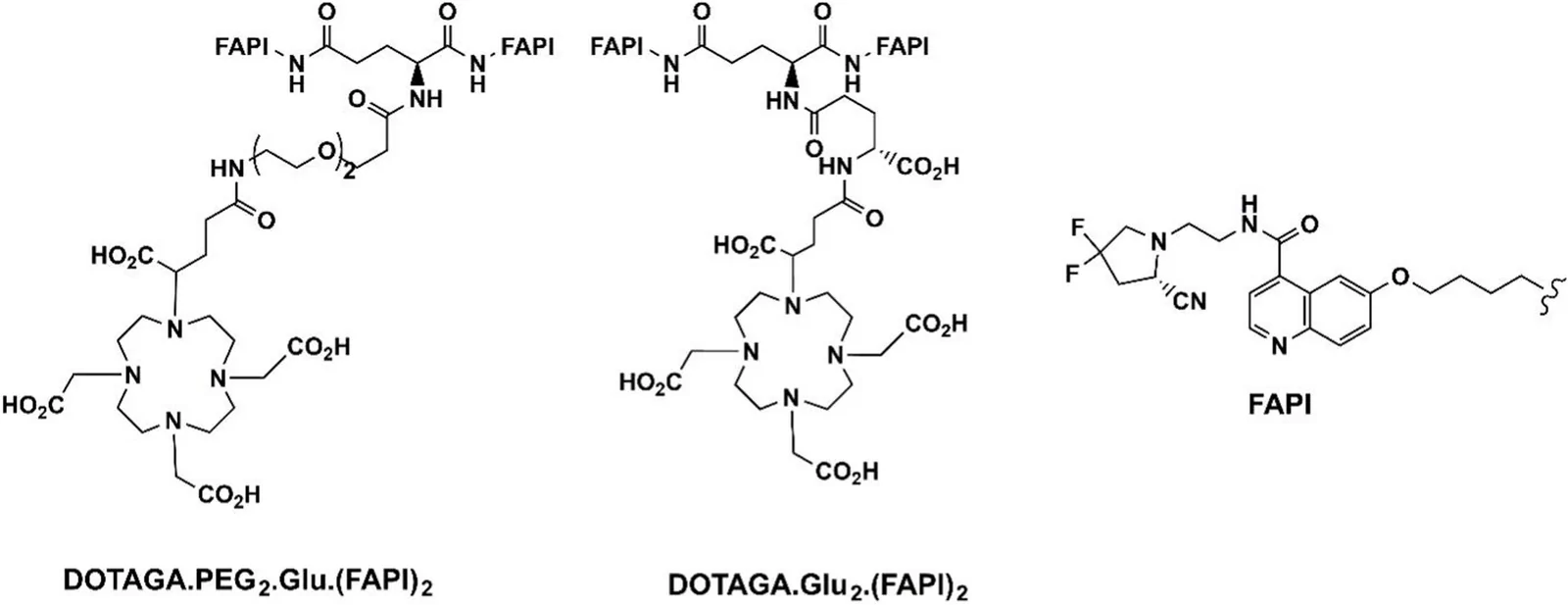
Schematic representations of DOTAGA.Glu_2_.(FAPI)_2_ and DOTAGA.PEG_2_.Glu.(FAPI)_2_

To overcome the limited tumor retention of monomeric FAPIs, several research groups have shifted toward the development of dimeric and multimeric constructs to improve pharmacokinetics and tumor residence time [13, 14, 16, 18–26]. In this context, our group advanced from the monomeric DOTA.SA.FAPI to the rational design of homodimeric derivatives [14–16, 21, 23]. The first-generation, DOTAGA.(SA.FAPI)_2_, and the second-generation homodimers, DOTAGA.Glu.(FAPI)_2_ and DO3A.Glu.(FAPI)_2_, demonstrated superior tumor uptake and prolonged retention compared to the monomeric analogue DOTA.SA.FAPI in preclinical and clinical studies [5–7, 14–17, 21, 23, 27]. Among these, DOTAGA.Glu.(FAPI)_2_ showed particularly promising performance [21]. To further improve tumor retention and elucidate the contribution of linker design to this property, the transition from the second- to the third-generation homodimers focused on the rational modification of the linker connecting the two FAPI moieties. As this linker critically influences molecular flexibility, hydrophilicity, and spatial orientation, and thus pharmacokinetics and tumor targeting, its careful redesign led to the development of the third-generation homodimers DOTAGA.Glu_2_.(FAPI)_2_ and DOTAGA.PEG_2_.Glu.(FAPI)_2_ (Fig. 1) [28]. Furthermore, their functionalization with DOTAGA enables stable radiolabeling with both ^68^Ga and ^177^Lu, facilitating a seamless transition from imaging to TRT.

Building on this rationale, we evaluated the biological performance and theranostic potential of DOTAGA.Glu_2_.(FAPI)_2_ and DOTAGA.PEG_2_.Glu.(FAPI)_2_, following radiolabeling with gallium-68 and lutetium-177. Particular emphasis was given on elucidating the impact of the linker composition on tumor targeting and pharmacokinetics to guide the development of optimized radiotherapeutics. In vitro studies assessed FAP-specific binding, internalization and externalization in FAP^+^ CAFs. In vivo evaluation in PC3-mice included biodistribution, PET/SPECT/CT imaging, metabolic stability, blood and organ clearance, complemented by ex vivo autoradiography. Finally, the therapeutic potential of [^177^Lu]Lu-DOTAGA.Glu_2_.(FAPI)_2_ was investigated using fractionated radioligand therapy, both as monotherapy and in combination with the mTOR inhibitor everolimus, to investigate potential synergistic effects through inhibition of tumor cell growth and radiosensitization.

## Materials and methods

### Radiolabeling / quality control / lipophilicity / protein binding studies

DOTAGA.Glu_2_.(FAPI)_2_ and DOTAGA.PEG_2_.Glu.(FAPI)_2_ (15 nmol each) were radiolabeled with gallium-68 using the Modular-Lab PharmTracer module (Eckert&Ziegler), and with lutetium-177 (5–20 nmol each) manually (Supplemental data). Radiochemical purity and stability (up to 168 h for the ^177^Lu-labelled compounds) were determined by reversed-phase high performance liquid chromatography (RP-HPLC) and radio thin layer chromatography (radio-TLC). The lipophilicity (LogD_octanol/PBS_) and protein binding were determined as described in the supplemental data.

### Cell lines

The human prostate adenocarcinoma cell line PC3 and the prostate-derived CAF cells were used in the present study. Cultivation conditions, materials and further details are described in the supplemental data.

### Saturation binding studies/internalization/externalization

For receptor saturation studies, FAP^+^ CAFs were incubated with 0.1 to 10 nM of the radioligands. For internalization studies, ~ 2.5 pmol of each radioligand was added and cells were incubated for 30, 60, 120, 240 and 360 min at 37 °C, 5% CO_2_. For externalization studies, cells were incubated with ~ 2.5 pmol of the ^177^Lu-labeled radioligands for 2 h at 37 °C, 5% CO_2_. The amount of externalized activity was measured at 0, 10, 20, 30, 60, 120, 240, and 1440 min (supplement).

### Animal models

Male athymic Balb/C nude mice (6 weeks/20–25 g) (RRID: IMSR_JAX:002019**)** were implanted with PC3 cells (3 × 10^6^/100 µL PBS) in the right shoulder to establish tumor model referred as PC3-mice. Biodistribution and PET/SPECT/CT studies were performed once tumors reached 250–300 mm^3^. For the therapy studies, treatment was initiated when the tumors reached ~ 100 mm^3^.

### Biodistribution studies

A total of 600 pmol (~ 4 MBq) of [^68^Ga]Ga-DOTAGA.Glu_2_.(FAPI)_2_ or [^68^Ga]Ga- DOTAGA.PEG_2_.Glu.(FAPI)_2_ in 100 µL of NaCl 0.9% was intravenously injected into PC3-mice, and biodistribution was assessed at 1, 2, and 3 h p.i. Similarly, 600 pmol (~ 6–7 MBq) of [^177^Lu]Lu-DOTAGA.Glu.(FAPI)_2_ or [^177^Lu]Lu-DOTAGA.PEG_2_.Glu.(FAPI)_2_ was administered, and biodistribution was evaluated at 4, 24, 48, 72, 96 and 168 h p.i. Blocking studies were performed by co-injection of 600 pmol of each radioligand with 20 nmol of the respective non-labeled ligand (total volume 100 µL) (supplement).

### Metabolic stability

Approximately 1 mL of blood from healthy mice was collected in heparinized tubes at 15 min p.i. of 600 pmol (~ 7 MBq) of [^177^Lu]Lu-DOTAGA.Glu_2_.(FAPI)_2_ or [^177^Lu]Lu-DOTAGA.PEG_2_.Glu.(FAPI)_2_ in 100 µL 0.9% NaCl. Blood samples were analyzed for potential metabolites by radio-HPLC (supplement).

### Blood and organs clearance kinetics

A total of 600 pmol (~ 7MBq) of [^177^Lu]Lu-DOTAGA.Glu_2_.(FAPI)_2_ or [^177^Lu]Lu-DOTAGA.PEG_2_.Glu.(FAPI)_2_ in 100 µL NaCl 0.9% was injected intravenously into healthy mice (*n* = 2). Blood samples were collected from the facial vein at various time points: 1, 3, 5, 7, 10, 15, 20, 30, 45, 60, 240, and till 5760 min p.i. The biological half-lives of the radioligands in various organs were calculated using the biodistribution data. Radioactivity was expressed as %I.A./g and plotted versus time (supplement).

### Area under the time-activity curves (AUC)

The area under the time-activity curves (AUC) for the tumor, salivary gland, liver, pancreas, bone and kidneys was calculated from the non-corrected biodistribution data using GraphPad Prism 10.2.0. AUC are expressed as % I.A./g×h and presented as mean values ± SD (*n* = 3).

### Small-animal PET/SPECT/CT

PET/CT scans were acquired on a micro-PET/SPECT/CT scanner (Albira Si; Bruker Biospin, Ettlingen, Germany). Static PET images were obtained 1, 2 and 3 h after intravenous injection of 600 pmol (~ 7 MBq/100 µL) of [^68^Ga]Ga**-**DOTAGA.Glu_2_.(FAPI)_2_ or [^68^Ga]Ga**-**DOTAGA.PEG_2_.Glu.(FAPI)_2_ in PC3-mice (*n* = 2). SPECT/CT images were acquired at 4, 24, 48, 72 and 96 h upon injection of 600 pmol (~ 14MBq/100 µL) of [^177^Lu]Lu-DOTAGA.Glu_2_.(FAPI)_2_ or [^177^Lu]Lu-DOTAGA.PEG_2_.Glu.(FAPI)_2_. For blocking studies, the PET/CT and SPECT/CT scans were acquired 1- and 4-hour p.i., respectively (supplement).

### Ex vivo autoradiography

Frozen tumor sections (7 μm) from PC3-mice injected with 600 pmol (~ 7MBq/100 µL) of [^68^Ga]Ga**-**DOTAGA.Glu_2_.(FAPI)_2_ or [^68^Ga]Ga**-**DOTAGA.PEG_2_.Glu.(FAPI)_2_ at 1 h p.i. were exposed to a Autoradiogrpahy Cassette FBCS 810 (FisherBiotech) for 1 h. Tumor sections from PC3-mice injected with the corresponding ^177^Lu-labeled radioligands were collected at 1.5 h p.i. and exposed overnight. The cassettes were scanned using a Typhoon 9400 analyzer (Amersham Biosciences/GE Healthcare) at 50 μm resolution. Images were analyzed with the OptiQuant software.

### Fractionated monotherapy with [^177^Lu]Lu-DOTAGA.Glu_2_.(FAPI)_2_

Tumor regression studies were conducted to assess the therapeutic effect of [^177^Lu]Lu-DOTAGA.Glu_2_.(FAPI)_2_ in PC3-mice using a fractionated dosing regimen. Once tumors reached ~ 100mm^3^ (108 ± 52mm^3^), mice were divided into three groups (*n* = 5/group). Two treatment groups received: (a) two injections of [^177^Lu]Lu-DOTAGA.Glu_2_.(FAPI)_2_ (~ 17–18 MBq, 600 pmol) on days 0 and 14 (total ~ 35 MBq, 1.2 nmol) and (b) four injections on days 0, 4, 14 and 18 (total ~ 70 MBq, 2.4 nmol). Control mice received PBS, following the same schedule (Figure S1). Tumor size and body weight were monitored three times weekly.

PET/CT imaging was performed to monitor therapy in all cohorts (*n* = 2) using [^68^Ga]Ga**-**DOTAGA.Glu_2_.(FAPI)_2_ (600 pmol, ~4MBq/100 µL) at 1 h p.i. on days 0, 11, 21, and 40 of treatment. Tumor volume was calculated as Width×(Length)^2^ × 0.5. Mice were euthanized if tumors exceeded 1.0 cm^3^, weight loss exceeded 15%, or at 70 days if endpoints were not reached.

### Combination therapy with [^177^Lu]Lu-DOTAGA.Glu_2_.(FAPI)_2_ and everolimus

The therapeutic efficacy of [^177^Lu]Lu-DOTAGA.Glu_2_.(FAPI)_2_ combined with everolimus was evaluated in PC3-mice (Figure S1) using the same tumor size criteria as above. Everolimus was selected for combination therapy based on its established role as an mTOR inhibitor in the treatment of prostate tumors and its potential to enhance the efficacy of targeted radionuclide therapy through inhibition of tumor cell growth and radiosensitization. Mice cohorts were (*n* = 5/group): (a) Everolimus alone (5 mg/kg/day) on days 0, 2, 4, 14, 16 and 18 and (b) Combination therapy with [^177^Lu]Lu-DOTAGA.Glu_2_.(FAPI)_2_ (~ 17–18 MBq, 600 pmol/dose; total: ~ 70 MBq, 2.4 nmol) on days 0, 4, 14 and 18 plus everolimus (5 mg/kg/day) on days 0, 2, 4, 14, 16 and 18. For comparison, the high-dose fractionated radioligand therapy cohort from the monotherapy study (four injections on days 0, 4, 14, and 18; total: ~70 MBq, 2.4 nmol) was used as the radionuclide therapy-only control group. Monotherapy and combination therapy studies were conducted in parallel and shared the same PBS control cohort. PET/CT imaging using [^68^Ga]Ga**-**DOTAGA.Glu_2_.(FAPI)_2_, tumor measurements, and body weight monitoring were performed according to the protocol described for the monotherapy study.

### Statistical analysis

Data are expressed as mean ± SD. Statistical analysis was performed using GraphPad Prism version 10.2.0 using the log-rank test; P˂ 0.05 was considered significant.

## Results

### Radiolabeling / quality control of the radioligands / stability

DOTAGA.Glu_2_.(FAPI)_2_ and DOTAGA.PEG_2_.Glu.(FAPI)_2_ (Fig. 1) were successfully radiolabeled with gallium-68 at > 99% radiochemical purity, with apparent molar activity (A_m_) of ~ 20 GBq/µmol (not decay-corrected). The ^177^Lu-labeling were almost quantitative. Both radioligands showed A_m_ values between 6 and 30 GBq/µmol, depending on the study. No colloid formation was observed. Quality control data are given in Figures S2, S3 (Supplement). The formulated ^177^Lu-labeled radioligands were highly stable, with no radiolysis or chemical decomposition detected over 4–168 h post labeling (Figure S4, S5).

### Lipophilicity / protein binding studies

LogD_octanol/PBS−pH7.4_ and human serum protein binding of the ^68^Ga- and ^177^Lu-labeled radioligands are depicted in Table 1. All radioligands were similarly hydrophilic with LogD ≤-3.

**Table 1:** LogD_octanol/PBS−pH7.4_, the percentage of human serum protein bound activity after 30 and 60 min of incubation

Tracer	LogD	Protein binding after 30 min	Protein binding after 60 min
[^68^Ga]Ga-DOTAGA.Glu_2_.(FAPI)_2_	-3.4 ± 0.3	11.3 ± 0.4	8.7 ± 0.4
[^177^Lu]Lu-DOTAGA.Glu_2_.(FAPI)_2_	-3.6 ± 0.3	6.2 ± 0.9	6.1 ± 0.4
[^68^Ga]Ga-DOTAGA.PEG_2_.Glu.(FAPI)_2_	-3.0 ± 0.3	8.4 ± 0.6	6.5 ± 0.3
[^177^Lu]Lu-DOTAGA.PEG_2_.Glu.(FAPI)_2_	-3.3 ± 0.1	4.2 ± 0.7	4.4 ± 0.3

[^68^Ga]Ga-DOTAGA.Glu_2_.(FAPI)_2_ and [^177^Lu]Lu-DOTAGA.Glu_2_.(FAPI)_2_ exhibited ~ 1.5-fold higher protein binding than [^68^Ga]Ga-DOTAGA.PEG_2_.Glu.(FAPI)_2_ and [^177^Lu]Lu-DOTAGA.PEG_2_.Glu.(FAPI)_2_ at 30 and 60 min of incubation with human serum.

### Saturation binding / internalization / externalization

**Fig. 2:**
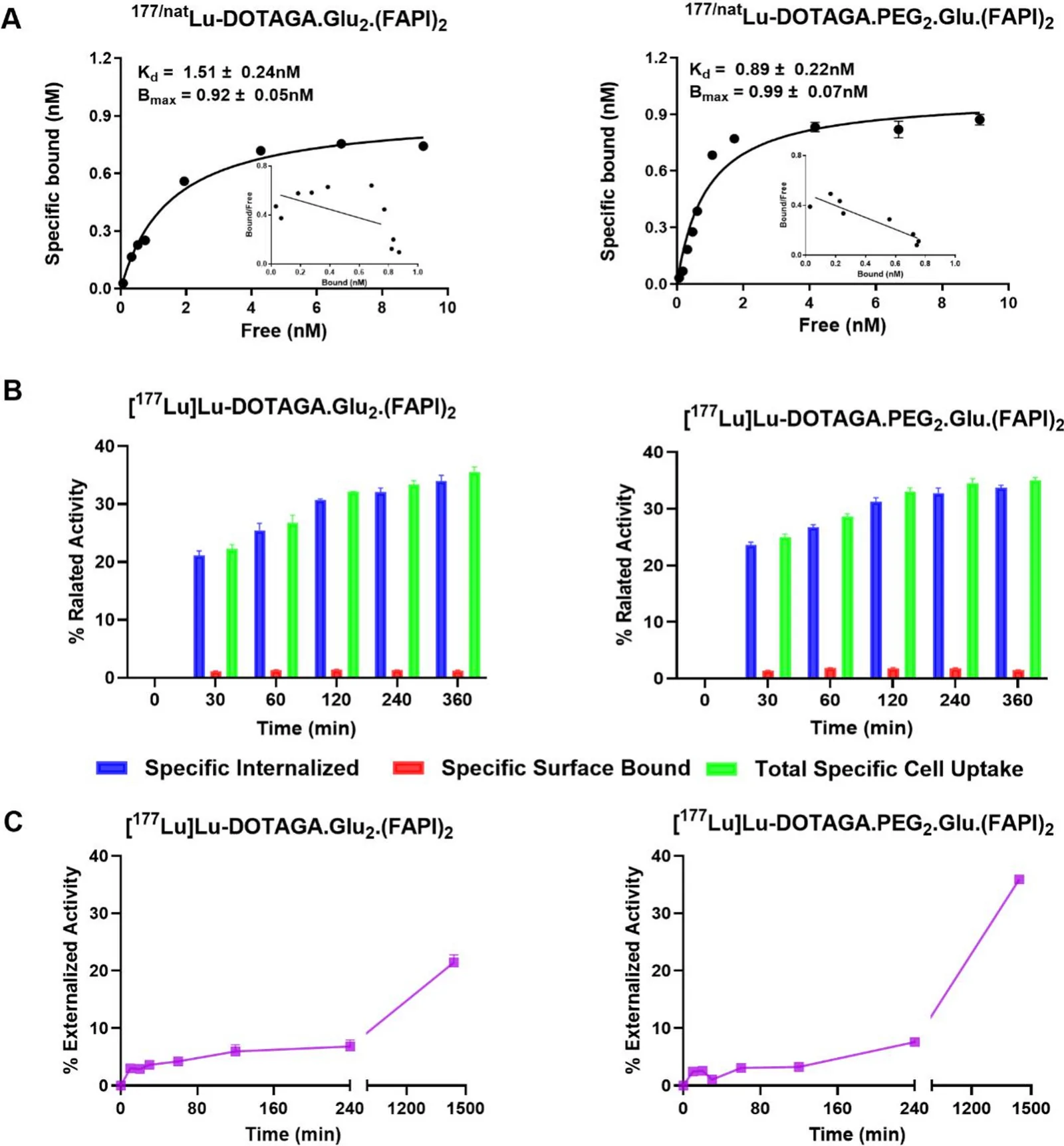
**A** Saturation binding on FAP^+^ CAF, using 0.1 to 10 nM of ^177/nat^Lu-DOTAGA.Glu_2_.(FAPI)_2_ and ^177/nat^Lu-DOTAGA.PEG_2_.Glu.(FAPI)_2_; Dissociation constant (K_d_) and B_max_ were calculated by nonlinear regression (GraphPad Prism 10.2.0). **B** Internalization and specific surface-bound fractions after incubation of CAFs with [^177^Lu]Lu-DOTAGA.Glu_2_.(FAPI)_2_ and [^177^Lu]Lu- DOTAGA.PEG_2_.Glu.(FAPI)_2_ at 37 °C; total specific cell uptake is the sum of both and expressed as % of the applied activity. Nonspecific binding was determined with 1 µM UAMC1110. **(C)** Dissociated activity expressed as % of the total cell-associated activity

^177/nat^Lu-DOTAGA.Glu_2_.(FAPI)_2_ and ^177/nat^Lu-DOTAGA.PEG_2_.Glu.(FAPI)_2_ exhibited high affinity for FAP^+^ CAFs (K_d_=1.5 ± 0.2 nM and 0.9 ± 0.2 nM, respectively) with similar B_max_ values (0.9-1.0 nM, ~6⋅10^5^ receptors/cell) (Fig. 2A). Both were internalized by CAFs with > 25% of the total added activity internalized after 60 min of incubation at 37 °C (Fig. 2B). [^177^Lu]Lu-DOTAGA.Glu_2_.(FAPI)_2_ showed higher cellular retention than [^177^Lu]Lu-DOTAGA.PEG_2_.Glu.(FAPI)_2_. After 24 h at 37 °C, ~ 20% of the initially cell-associated activity was released from the CAFs for [^177^Lu]Lu-DOTAGA.Glu_2_.(FAPI)_2_, compared with ~ 35% for [^177^Lu]Lu- DOTAGA.PEG_2_.Glu.(FAPI)_2_ (Fig. 2C). Blocking with UAMC1110 (1 µM), confirmed high specificity of both radioligands towards FAP^+^ CAFs.

### Biodistribution studies

**Fig. 3:**
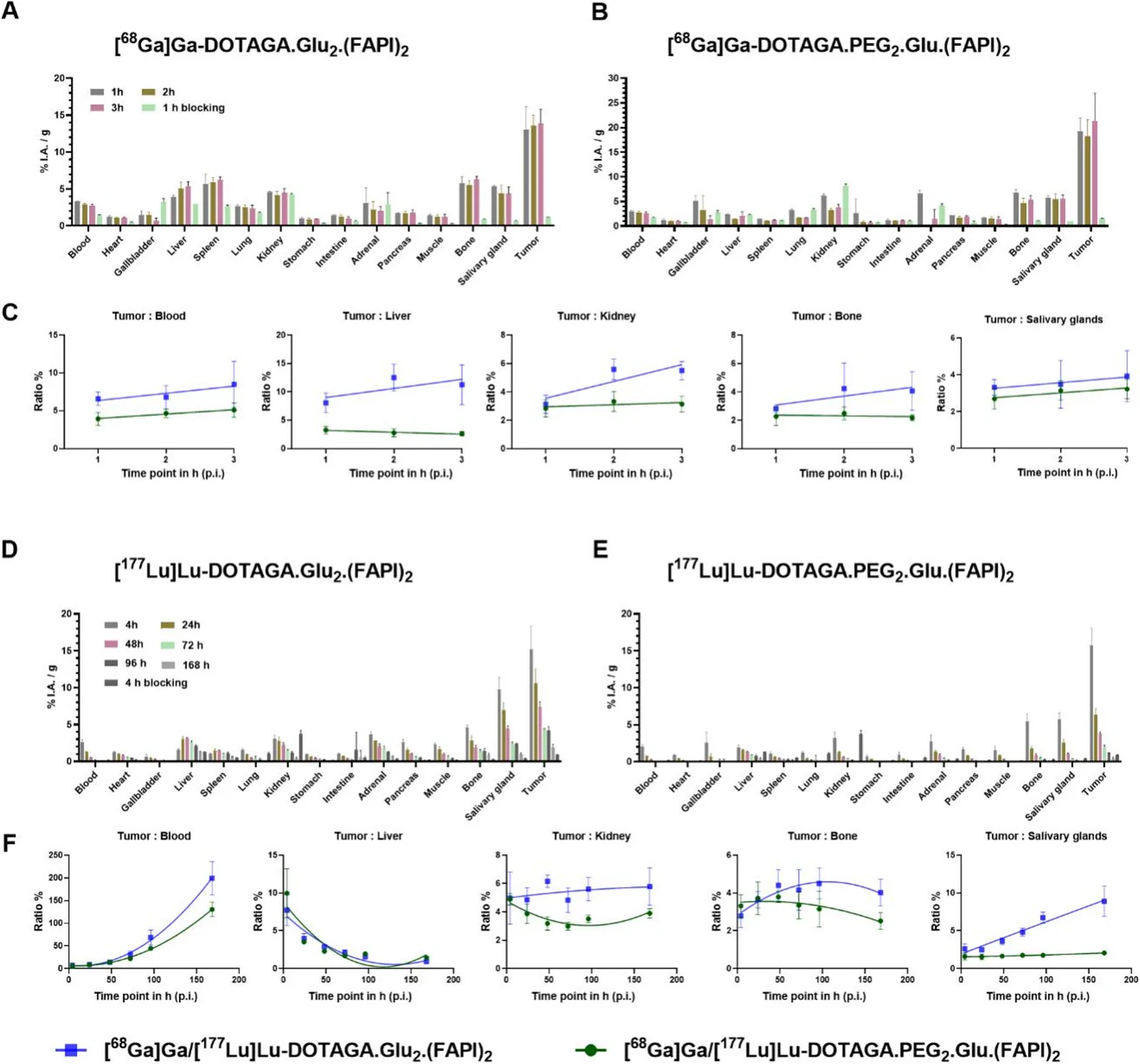
Biodistribution of (**A**) [^68^Ga]Ga-DOTAGA.Glu_2_.(FAPI)_2_ and (**B**) [^68^Ga]Ga-DOTAGA.PEG_2_.Glu.(FAPI)_2_ in PC3-mice at 1, 2, and 3 h p.i, including 1 h blocking data. **C** Tumor-to-background ratios for [^68^Ga]Ga-DOTAGA.Glu_2_.(FAPI)_2_ and [^68^Ga]Ga-DOTAGA.PEG_2_.Glu.(FAPI)_2_. **D** Biodistribution of [^177^Lu]Lu-DOTAGA.Glu_2_.(FAPI)_2_ and (**E**) [^177^Lu]Lu-DOTAGA.PEG_2_.Glu.(FAPI)_2_ at 4, 24, 48, 72, 96 and 168 h p.i. with 4 h blocking data. **F** Tumor-to-background ratios of [^177^Lu]Lu-DOTAGA.Glu_2_.(FAPI)_2_ and [^177^Lu]Lu-DOTAGA.PEG_2_.Glu.(FAPI)_2_. All biodistribution data have been calculated as % I.A./g of tissue and are presented as mean ± SD (*n* = 3)

Both ^68^Ga-labeled FAPI-homodimers showed high and consistent tumor uptake over 3 h p.i. (~ 13.5%I.A./g for [^68^Ga]Ga-DOTAGA.Glu_2_.(FAPI)_2_ vs. ~ 20%I.A./g for [^68^Ga]Ga-DOTAGA.PEG_2_.Glu.(FAPI)_2_) and low blood retention (2.5-3%I.A./g, 3 h p.i.). [^68^Ga]Ga-DOTAGA.Glu_2_.(FAPI)_2_ exhibited higher liver uptake than its analogue DOTAGA.PEG_2_.Glu.(FAPI)_2_ (*p* < 0.01), whereas kidney uptake was similar. Bone and salivary gland uptake likely reflect their physiological FAP expression (Fig. 3A–C and Tables S1, S2).

The ^177^Lu-labelled radioligands showed similar initial tumor uptake (~ 15 %I.A./g at 4 h p.i.), consistent with their ^68^Ga-labeled analogs. However, tumor retention differed over time: tumor uptake of [^177^Lu]Lu-DOTAGA.PEG_2_.Glu.(FAPI)_2_ decreased from 6.4 ± 0.8%I.A./g at 24 h p.i. to 3.7 ± 0.3%I.A./g at 48 h p.i., whereas [^177^Lu]Lu-DOTAGA.Glu_2_.(FAPI)_2_ showed significantly higher retention of 10.6 ± 1.9%I.A./g and 7.3 ± 0.7%I.A./g at the same time points. This difference persisted over 96 and 168 h (96 h p.i.: 4.2 ± 0.5 vs. 1.2 ± 0.02; 168 h p.i.: 2.0 ± 0.4 vs. 0.4 ± 0.2%I.A./g for the Glu_2_ vs. the PEG_2_Glu derivative, respectively). Background uptake was low and comparable for both, with elevated salivary gland and bone uptake likely due to physiological FAP expression (Figs. 3D–F and Tables S3, S4).

### Metabolic stability / Blood and organs clearance / AUC of [^177^Lu]Lu-DOTAGA.Glu_2_.(FAPI)_2_ and [^177^Lu]Lu-DOTAGA.PEG_2_.Glu.(FAPI)_2_

**Fig. 4:**
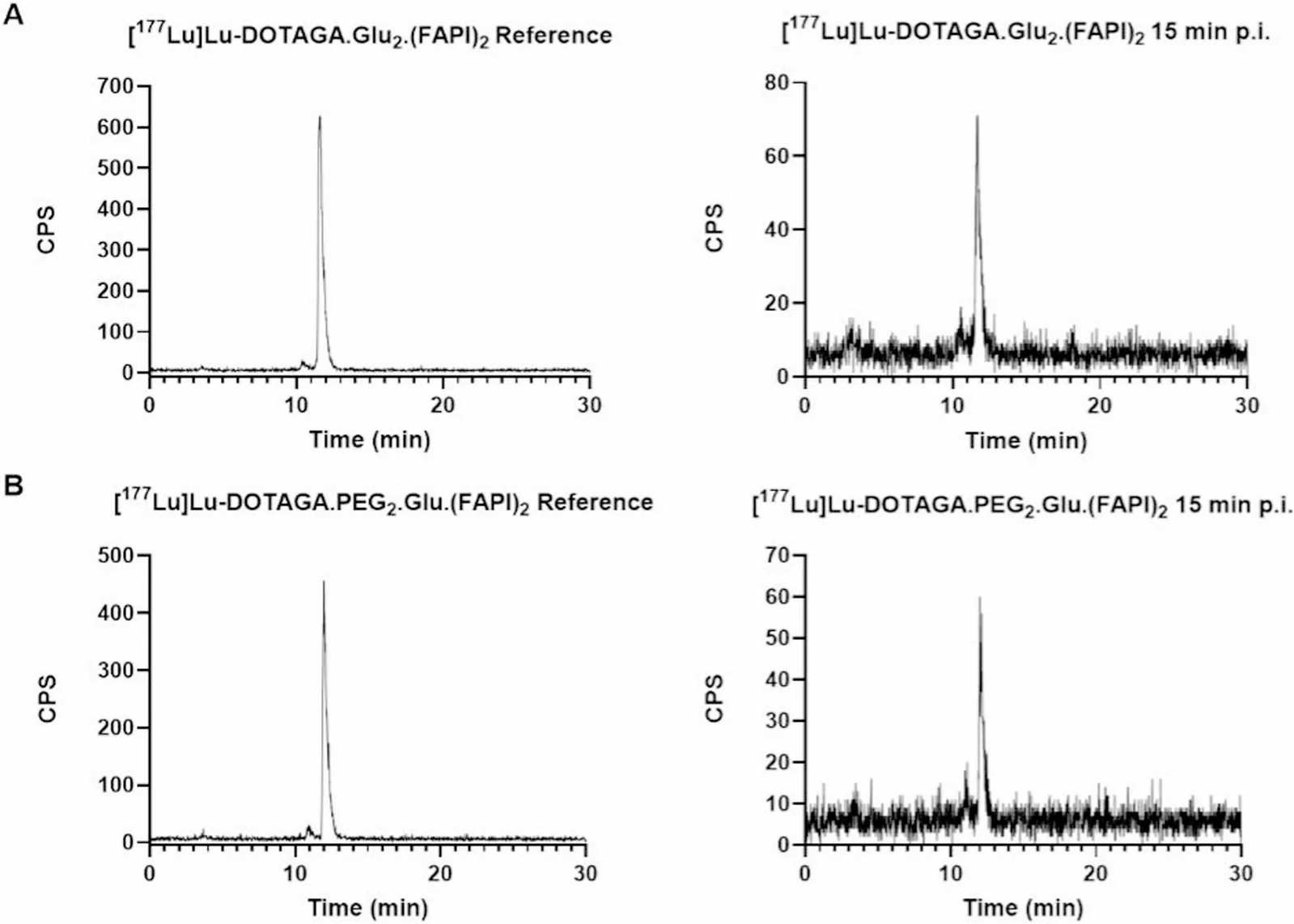
Representative radio-HPLC chromatograms (**A**) [^177^Lu]Lu-DOTAGA.Glu_2_.(FAPI)_2_ and (**B**) [^177^Lu]Lu-DOTAGA.PEG_2_.Glu.(FAPI)_2_. Left: reference chromatograms before injection; right: plasma samples collected 15 min p.i

HPLC analysis of mouse plasma showed only radioligands in circulation 15 min p.i. (Fig. 4A, B).

**Fig. 5:**
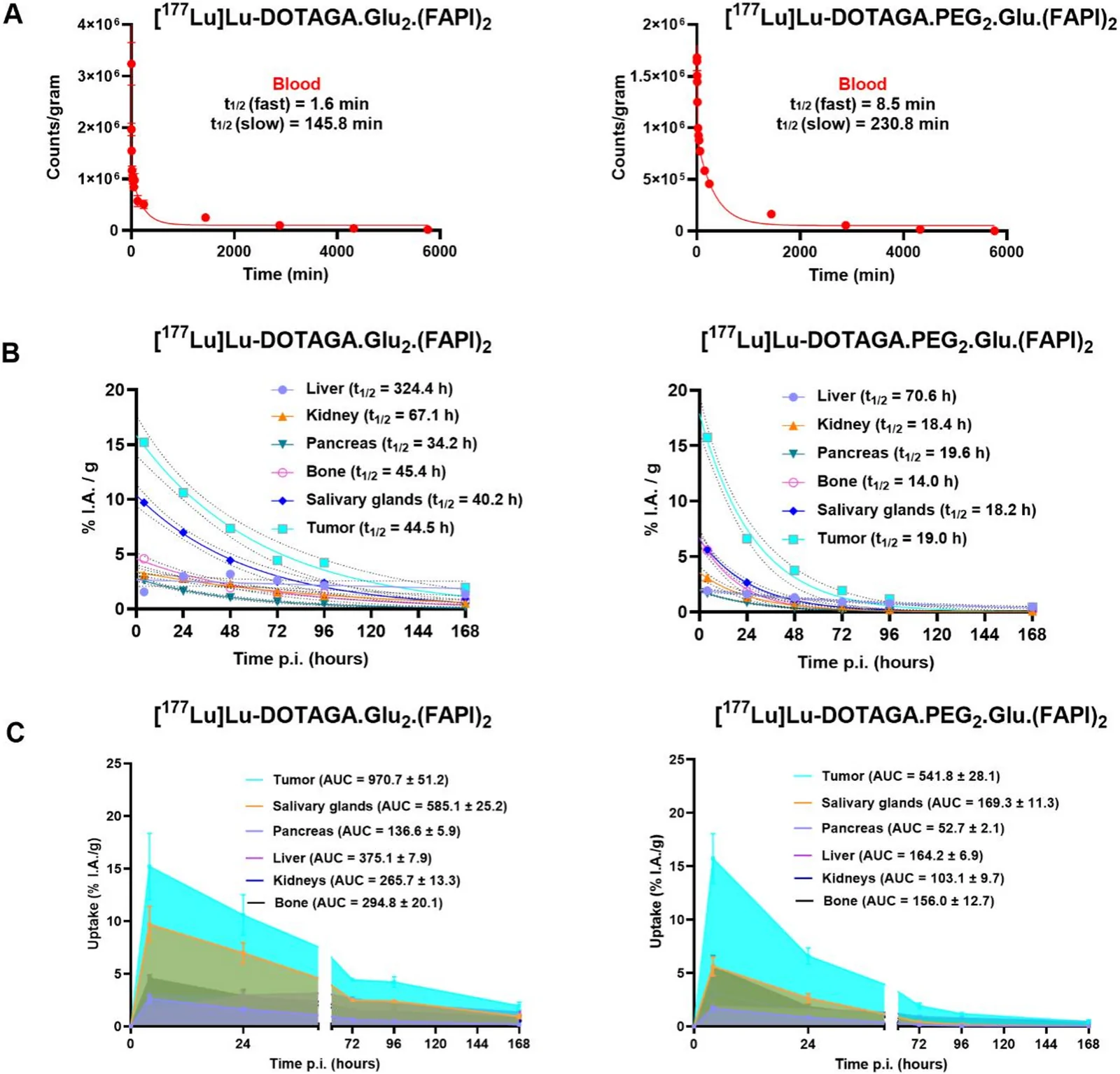
**A** Blood clearance of [^177^Lu]Lu-DOTAGA.Glu_2_.(FAPI)_2_ and [^177^Lu]Lu-DOTAGA.PEG_2_.Glu.(FAPI)_2_ over time. **B** Organ clearance kinetics. **C** Area under the time activity curve (AUC) for tumor, salivary glands, pancreas, liver, kidneys and bone, calculated from biodistribution data and expressed as % I.A./g×h ± SD

Blood clearance (Fig. 5A) was rapid and biphasic for both, with slightly faster clearance for [^177^Lu]Lu-DOTAGA.Glu_2_.(FAPI)_2_ (t_1/2_: 1.6 and 145.8 min), compared to [^177^Lu]Lu-DOTAGA.PEG_2_.Glu.(FAPI)_2_ (t_1/2_: 8.5 and 230.8 min).

Organ kinetics (Fig. 5B and Table S5) demonstrated substantially prolonged tumor retention for [^177^Lu]Lu-DOTAGA.Glu_2_.(FAPI)_2_ (t_1/2_: 44 vs. 19 h), while non-target clearance was slower for [^177^Lu]Lu-DOTAGA.Glu_2_.(FAPI)_2_ across major organs, including liver, kidneys, pancreas, bone, and salivary glands.

AUC analysis (Fig. 5C) further confirmed higher tumor exposure for [^177^Lu]Lu-DOTAGA.Glu_2_.(FAPI)_2_ compared to [^177^Lu]Lu-DOTAGA.PEG_2_.Glu.(FAPI)_2_ (971 ± 74 vs. 542 ± 28%I.A./g×h), corresponding to ~ 2-fold increase.

### Small-animal PET/SPECT/CT

**Fig. 6:**
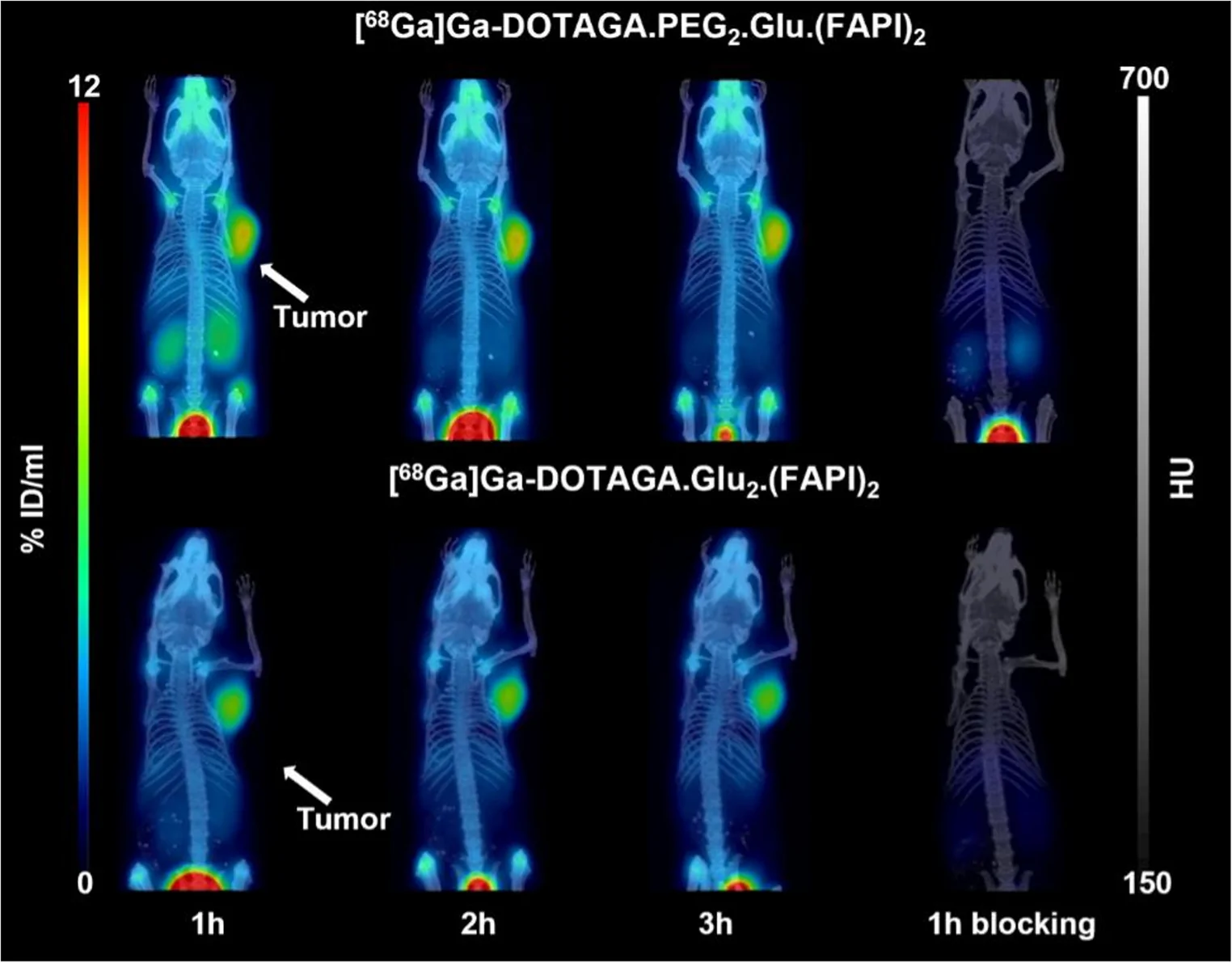
PET/CT images of [^68^Ga]Ga-DOTAGA.PEG_2_.Glu.(FAPI)_2_ and [^68^Ga]Ga-DOTAGA.Glu_2_.(FAPI)_2_ in PC3-mice at 1, 2, and 3 h p.i. with 1 h blocking

PET/CT images of PC3-mice (Fig. 6) clearly demonstrated consistent tumor retention at all time points with low background, in agreement with the biodistribution data.

**Fig. 7:**
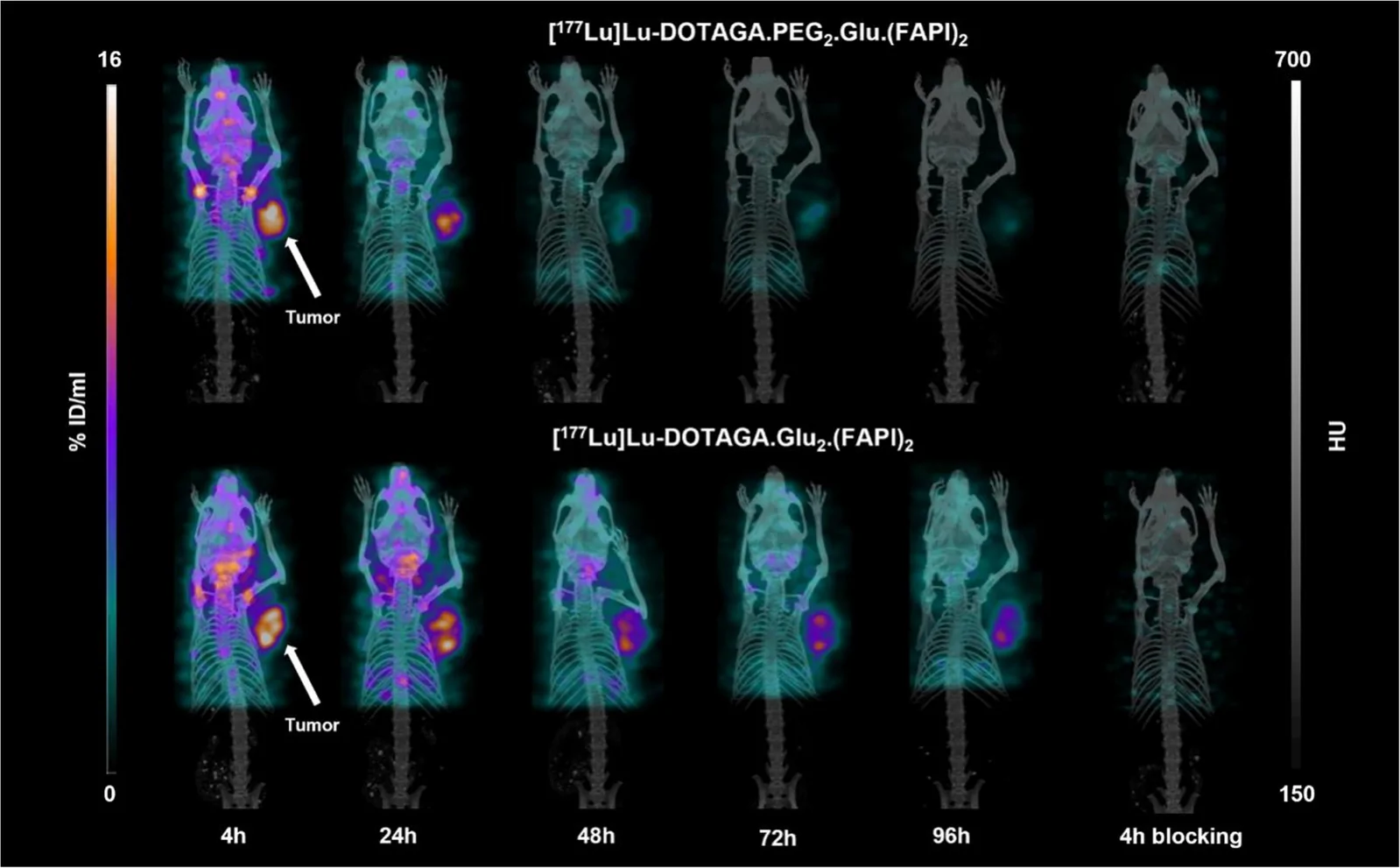
SPECT/CT images of [^177^Lu]Lu-DOTAGA.Glu_2_.(FAPI)_2_ and [^177^Lu]Lu-DOTAGA.PEG_2_.Glu.(FAPI)_2_ in PC3-mice at 4, 24, 48, 72 and 96 h p.i., with 4 h blocking

The corresponding SPECT/CT images (Fig. 7), confirmed these findings, with slightly higher background at 4 h p.i., particularly in bone and salivary glands. Tumor uptake was evident at all time points, highlighting the faster washout of [^177^Lu]Lu-DOTAGA.PEG_2_.Glu.(FAPI)_2_ compared to [^177^Lu]Lu-DOTAGA.Glu_2_.(FAPI)_2_, particularly at later time points. Blocking studies confirmed specific binding of all radioligands.

#### Ex vivo autoradiography

**Fig. 8:**
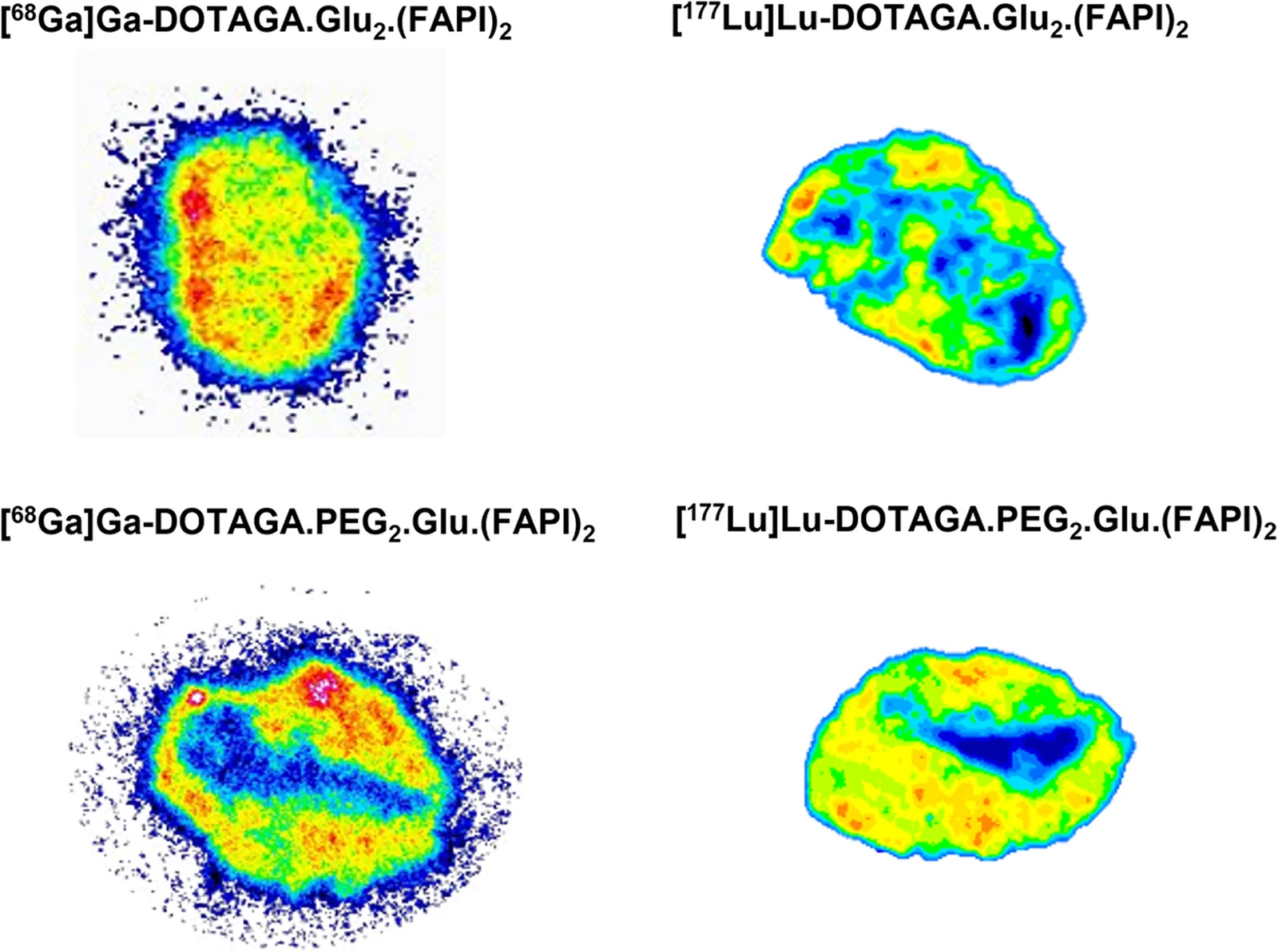
Ex vivo autoradiography of PC3-tumor sections after injection of [^68^Ga]Ga-DOTAGA.PEG_2_.Glu.(FAPI)_2_, [^177^Lu]Lu-DOTAGA.PEG_2_.Glu.(FAPI)_2_, [^68^Ga]Ga-DOTAGA.Glu_2_.(FAPI)_2_ and [^177^Lu]Lu-DOTAGA.Glu_2_.(FAPI)_2_

Autoradiography of tumor sections indicated heterogeneous radioactivity distribution in PC3-tumors (Fig. 8), predominantly localized at the tumor periphery.

### Fractionated monotherapy with [^177^Lu]Lu-DOTAGA.Glu_2_.(FAPI)_2_

**Fig. 9:**
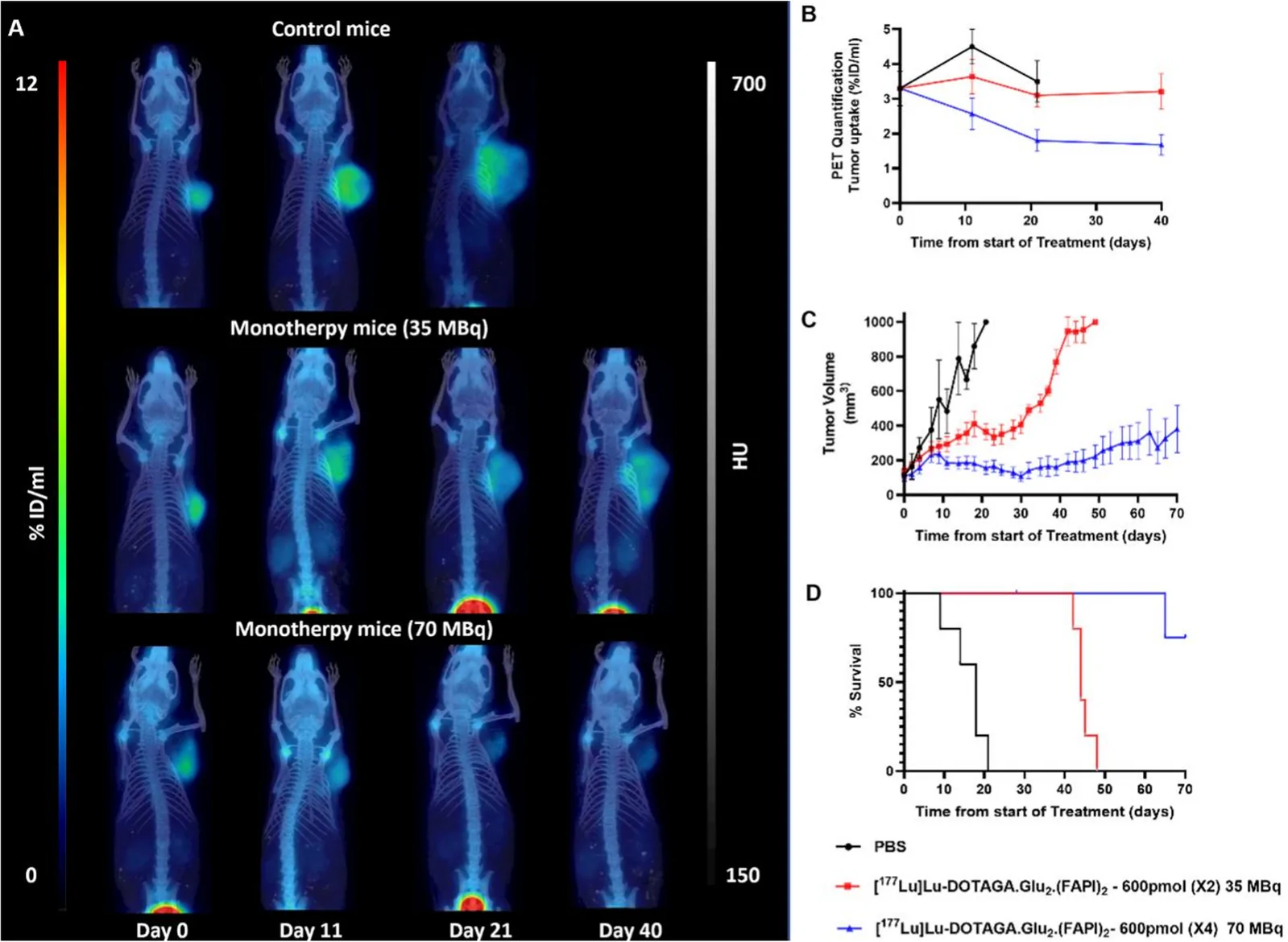
Monotherapy study (**A**) Static PET/CT scans with [^68^Ga]Ga-DOTAGA.Glu_2_.(FAPI)_2_ (1 h p.i., ~ 7 MBq; *n* = 2) on days 0, 11, 21 and 40. **B** PET-based tumor uptake quantification. **C** Tumor volume (mm^3^) over the treatment period. **D** Survival curves of the different monotherapy groups

Control tumors (PBS) progressed rapidly, with all mice reaching endpoints within 21 days. The low-dose group (35 MBq, 2 doses) showed improved tumor control, reaching endpoints within 49 days. The high-dose group (70 MBq, 4 doses) exhibited the strongest suppression, maintaining tumor volumes < 400 mm^3^ until day 70. Survival reflected these trends (18 vs. 44 days for PBS and the low-dose (*p* < 0.01); >50% survival in the high-dose at study end (one mouse initially responded but died unexpectedly on day 28 (tumor-volume 157 mm^3^), one reached the tumor- endpoint on day 65, while three mice remained endpoint-free until day 70 (380 ± 138 mm^3^)), with stable body weight (Fig. 9).

PET imaging during the therapy using [^68^Ga]Ga-DOTAGA.Glu_2_.(FAPI)_2_ showed increasing tumor uptake in controls of 3.3 to 4.5%ID/mL by day 11, then declining to 3.5%ID/mL most probably due to necrosis. The low-dose group exhibited only a slight increase from 3.3%ID/mL (day 0) to 3.6%ID/mL (day 11), and remained below controls, while the high-dose group showed a marked decrease from 3.3%ID/mL to 1.7%ID/mL (day 0 and day 40, respectively), about 2-fold lower than the low-dose group.

### Combination therapy with [^177^Lu]Lu-DOTAGA.Glu_2_.(FAPI)_2_ and everolimus

**Fig. 10:**
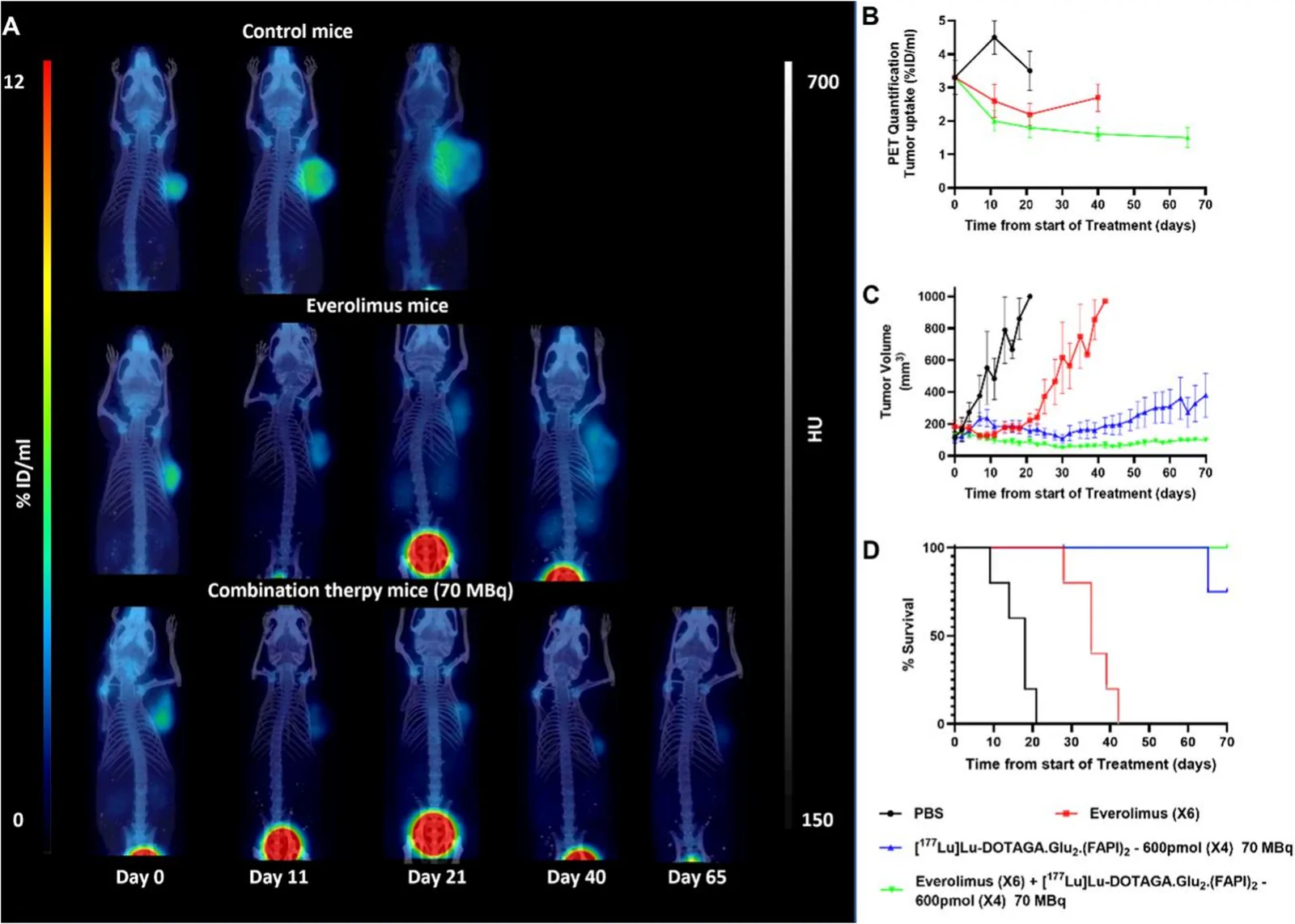
Combination therapy study (**A**) Static PET scans of [^68^Ga]Ga-DOTAGA.Glu_2_.(FAPI)_2_ (1 h p.i., ~ 7 MBq; *n* = 2) on days 0, 11, 21, 40 and 65. **B** PET-based tumor uptake quantification. **C** Tumor volume (mm^3^) over the treatment period. **D** Survival curves of the combination therapy groups. Note: The PET images and quantitative PET data shown for the radioligand monotherapy group are identical to those of the high-dose monotherapy cohort presented in Fig. 9A and B, as this cohort was used as the radioligand-only comparator in the combination therapy study

PBS controls showed rapid tumor growth (> 800 mm^3^ by day 15), while everolimus alone moderately delayed growth (< 500 mm^3^ for 30 days). Radioligand monotherapy further improved tumor-control (< 200 mm^3^ for 45 days; <400 mm^3^ until day 70). The combination therapy was most effective, maintaining tumor volumes < 100 mm^3^ for 70 days, indicating a strong synergistic effect (Fig. 10). Survival analysis confirmed this benefit, with 100% survival in the combination group vs. 50% for radioligand alone; median survival was ~ 18 and ~ 34 days for control and everolimus groups (*p* < 0.01).

PET monitoring using [^68^Ga]Ga-DOTAGA.Glu_2_.(FAPI)_2_ and consequently PET quantification demonstrated distinct treatment-dependent changes in tumor uptake. In the everolimus-only group, tumor uptake gradually decreased from 3.3%ID/mL (day 0) to 2.2%ID/mL (day 21), followed by a non-significant rise to 2.7%ID/mL at day 40 (*p* > 0.5). In the radioligand monotherapy cohort, previously shown in the fractionated monotherapy study, uptake progressively declined from 3.3%ID/mL at baseline to 1.7%ID/mL by day 40, consistent with sustained tumor suppression. The combination therapy produced the strongest response, with tracer uptake decreasing further to 1.6%ID/mL by day 65, supporting the enhanced therapeutic efficacy observed in tumor growth inhibition and survival analyses.

All treatments (monotherapy and combination therapy) were well tolerated, with no observable side effects.

## Discussion

FAP-targeting radiopharmaceuticals have rapidly evolved from monomeric FAPI ligands toward more advanced constructs designed to overcome the key limitation of short tumor retention [9, 11, 13–16, 18–26, 29, 30]. While monomeric FAPIs provide excellent imaging contrast, their fast washout restricts their therapeutic applicability, particularly when labeled with β-emitting radionuclides. To address this, several strategies have been explored, including peptide-based tracers (FAP-2286, FAP-HXN), albumin-binding modifications (FAPI-46-EB, TEFAPI-06/07), and multivalent approaches such as tetrameric constructs (DOTA-4P(FAPI)_4_) (Table S6) [13, 26, 29, 30]. Although these have improved tumor residence time, they are often associated with increased off-target accumulation or model-dependent behavior (Table S6). Among these, dimerization has emerged as a promising approach to enhance tumor retention through increased avidity and optimized pharmacokinetics [14, 20–25].

Building on our initial work with the monomer DOTA.SA.FAPI, which revealed suboptimal pharmacokinetic behavior when intended for therapeutic applications, our group pursued a systematic development of FAPI homodimers [5–8, 14–16, 21, 23]. The first-generation homodimer, DOTAGA.(SA.FAPI)_2_, demonstrated improved tumor targeting but still showed limitations related to lipophilicity, protein binding and notable liver uptake [14–16]. These limitations were also evident in clinical settings, highlighting the need for further optimization [6, 7, 17]. This led to the development of the second generation homodimers DOTAGA.Glu.(FAPI)_2_ and DO3A.Glu.(FAPI)_2_ [21, 23]. A major improvement was achieved with DOTAGA.Glu.(FAPi)_2_, where substitution of the squaric acid with the glutamic acid linker, together with a more favorable positioning of the chelator relative to the FAPI pharmacophore, improved the overall molecular architecture [21]. In particular, distancing the bulky radiometal-chelator complex from the FAPI binding motif reduced steric interference and improved target accessibility. At the same time, increased polarity reduced nonspecific interactions, resulting in enhanced hydrophilicity, lower protein binding, and improved pharmacokinetics while maintaining high FAP affinity.

The present study extends this concept to the third-generation of homodimers, DOTAGA.(Glu)_2_.(FAPI)_2_ and DOTAGA.PEG_2_.Glu.(FAPI)_2_, designed to further fine-tune the spatial arrangement and physicochemical properties of the dimers [28]. Specifically, the introduction of a second glutamic acid for DOTAGA.(Glu)_2_.(FAPI)_2_ was intended to increase molecular rigidity, negative charge, and hydrophilicity, thereby reducing nonspecific binding and promoting prolonged tumor retention. In the case of DOTAGA.PEG_2_.Glu.(FAPI)_2_, the PEG_2_ linker was selected to introduce greater flexibility and spatial separation between the two FAPI units, potentially facilitating simultaneous or independent interactions with FAP while maintaining low steric hindrance. Thus, the two designs represent complementary strategies: a more rigid, polar scaffold for DOTAGA.(Glu)_2_.(FAPI)_2_ versus a more flexible, hydrophilic spacer for DOTAGA.PEG_2_.Glu.(FAPI)_2_, enabling a direct assessment of how linker composition directs the in vivo behavior of the derived radiopharmaceuticals.

Both theranostic pairs retained high affinity and specificity toward FAP, confirming that these structural modifications do not compromise target binding (Table S7). However, clear differences were observed in vivo. For these studies, the administered mass was carefully selected based on our prior dose-escalation experiments in PC3-mice, which demonstrated a clear mass-dependent effect on biodistribution and overall pharmacokinetic performance [15, 16, 21]. An intermediate dose of 600 pmol, previously identified by our group as the optimal injected mass for FAPI-based homodimers, was used in all experiments to ensure sufficient receptor engagement while avoiding saturation and increased background signal [15, 16, 21]. This approach ensures that the observed differences are attributable to structural variations rather than dosing effects. While initial tumor uptake was comparable for all the tested radioligands, the (Glu)_2_-based homodimer showed significantly prolonged tumor retention, particularly evident in the head-to-head comparison of the therapeutic radioligands, whereas the PEG_2_.Glu-based analogue exhibited faster washout. This is consistent with the in vitro data, where enhanced cellular retention and reduced externalization were also observed for the (Glu)_2_-based construct. Autoradiography further revealed a heterogeneous intratumoral distribution of radioactivity, with predominant localization at the tumor periphery. This pattern likely reflects the uneven distribution of FAP-expressing CAFs and differences in vascularization within PC3-tumors, where peripheral regions are generally better perfused and more stromal-rich, facilitating radioligand delivery and retention, whereas central regions may contain hypoxic or necrotic areas with reduced radioligand penetration. Such non-uniform uptake may result in reduced radiation delivery to poorly vascularized or stromal-poor tumor areas, potentially limiting treatment efficacy. However, the longer tissue penetration range of β-emitting radionuclides, such as lutetium-177, may partially compensate for heterogeneous uptake through cross-fire irradiation of adjacent tumor regions. In contrast, α-emitting radionuclides, owing to their substantially shorter path length, may require more homogeneous intratumoral localization to achieve optimal therapeutic effects. Furthermore, these findings support the potential value of combination treatment strategies aimed at improving tumor coverage and overall therapeutic efficacy.

Our data suggest that increased rigidity and polarity may contribute to sustained tumor localization, whereas higher flexibility may facilitate faster dissociation and clearance. Importantly, the improved tumor retention of the (Glu)_2_-based homodimer was achieved without a substantial increase in off-target accumulation. Although a moderate increase in liver uptake was observed, the overall background activity remained low, preserving favorable tumor-to-background ratios. Compared with alternative strategies such as albumin-binding or higher-order of multimerization, which often increase systemic exposure, the homodimer approach enables a more balanced optimization of pharmacokinetics through rational structural design (Table S6).

A key finding of this study is the clear demonstration of the therapeutic efficacy using fractionated administration of [^177^Lu]Lu-DOTAGA.Glu_2_.(FAPI)_2_. Fractionated regimens enable repeated radiation delivery while allowing recovery of normal tissues, thereby increasing cumulative tumor dose without exceeding toxicity limits in critical organs such as bone marrow and kidneys [31]. This approach is particularly relevant for FAPI-based radioligands, which are often limited by suboptimal tumor retention. Particularly, clinical studies employing repeated cycles of FAPI radioligand therapy have already indicated good tolerability and signs of therapeutic benefit [31]. In line with these observations, fractionated dosing of [^177^Lu]Lu-DOTAGA.Glu_2_.(FAPI)_2_ in the present study resulted in sustained tumor control, significant tumor growth inhibition, and a pronounced survival benefit compared to the control groups, highlighting its strong therapeutic potential.

We further explored a combination strategy based on complementary targeting of distinct tumor compartments [32–34]. While FAP-targeted radionuclide therapy primarily addresses the tumor stroma, mTOR inhibition with everolimus directly targets tumor cells by interfering with key pathways regulating proliferation, metabolism, and survival [4, 35–37]. We therefore hypothesized that simultaneous targeting of stromal and malignant components would enhance overall therapeutic efficacy. Consistent with this rationale, the combination therapy outperformed both monotherapies, resulting in superior tumor control and improved survival. In our PC3 tumor model, the combination of [^177^Lu]Lu-DOTAGA.Glu_2_.(FAPI)_2_ with everolimus maintained tumor volumes below 100 mm^3^ for up to 70 days and achieved 100% survival, whereas both monotherapies showed only partial therapeutic benefit. Comparable enhancement of therapeutic efficacy through combinatorial approaches has also been reported for other FAP-targeted radioligands in combination with immune checkpoint inhibition (Table S8) [32–34]. For example, the combination of [^177^Lu]Lu-FAP-2287 with anti-PD-1 therapy prolonged median survival compared with either monotherapy in MCA205-mFAP tumors [33], while combinations of [^177^Lu]Lu-DOTA-2P(FAPI)_2_ or [^177^Lu]Lu-LNC1004 with PD-1/PD-L1 blockade resulted in markedly improved tumor control and complete or near-complete long-term survival in CT26-FAP and MC38/NIH3T3-FAP tumor models, respectively [32, 34]. Although these studies employed different tumor models and therapeutic strategies, together with our findings they support the concept that multi-compartment targeting can overcome limitations of single-modality approaches and substantially enhance the efficacy of FAP-targeted radionuclide therapy. Our data further extend this concept by demonstrating that combination with mTOR inhibition represents an effective non-immunotherapeutic strategy for potentiating FAP-targeted TRT.

Importantly, therapy monitoring, using the diagnostic counterpart [^68^Ga]Ga-DOTAGA.(Glu)_2_.(FAPI)_2_-PET, provided real-time, non-invasive insight into the therapeutic response. Changes in radiotracer uptake closely paralleled treatment efficacy, confirming that PET imaging can serve as a reliable biomarker for monitoring FAP-targeted therapies and guiding treatment strategies. This integrated theranostic approach demonstrates the translational potential of the third-generation of FAPI homodimers, combining high-contrast imaging with effective targeted radionuclide therapy.

Since FAP expression and stromal composition vary considerably across tumor entities, the observed biodistribution, tumor retention, and therapeutic effects in PC3-mice may not necessarily be representative of other tumor types and this will be the important direction for future investigation in additional preclinical models.

In summary, the progressive optimization from first- to third-generation FAPI homodimers demonstrates how rational structural refinement can substantially improve pharmacokinetics, tumor retention, and therapeutic performance. These findings highlight the importance of linker design for balancing tumor uptake, retention, and background clearance, and support the continued development of optimized FAPI homodimers for theranostic applications in FAP-expressing malignancies.

## Conclusion

This study identifies DOTAGA.(Glu)_2_.(FAPI)_2_ as the most promising FAPI homodimer, combining high FAP affinity, prolonged tumor retention, favorable pharmacokinetics, and strong therapeutic efficacy. [^177^Lu]Lu-DOTAGA.(Glu)_2_.(FAPI)_2_ achieved effective tumor control under fractionated dosing and showed additional benefit in combination with everolimus, while [^68^Ga]Ga-DOTAGA.(Glu)_2_.(FAPI)_2_ enabled non-invasive therapy monitoring. Together, these findings support the clinical translation of structurally optimized FAPI homodimers as theranostic agents for FAP-expressing tumors.

## Supplementary Information

Below is the link to the electronic supplementary material.

Supplementary Material 1

## Data Availability

The datasets generated during and/or analyzed during the current study are available from the corresponding author on reasonable request.
