# Supplementary material for: Optimizing FAPI homodimers for theranostic applications: The impact of the linker design in DOTAGA.Glu2.(FAPI)2 and DOTAGA.PEG2.Glu.(FAPI)2
Source: Eur J Nucl Med Mol Imaging. 2026 Jul 15;53(12):7093–108. doi: 10.1007/s00259-026-08078-9 (PMC13633386; doi:10.1007/s00259-026-08078-9)
Supplement: Supplementary file 1 [file 259_2026_8078_MOESM1_ESM.docx]

**Supplemental Data**

**Optimizing FAPI Homodimers for Theranostics: The Impact of the Linker Design in DOTAGA.Glu_2_.(FAPI)_2_ and DOTAGA.PEG_2_.Glu.(FAPI)_2_**

Naveen Kumar^1,2^, Adrianna Bilinska^1,2,3^, Elena Menéndez^1,2^, Sarah Stephan^4^, Frank Rösch^4^, Axel Rominger^1,2^, Eleni Gourni^1,2^

^1^Department of Nuclear Medicine, Inselspital, University Hospital Bern, Bern, Switzerland,

^2^Department of Biomedical Research, University of Bern, Bern, Switzerland

^3^Graduate School of Cellular and Biomedical Sciences, University of Bern, Bern, Switzerland

^4^Department of Chemistry–TRIGA site, Johannes Gutenberg-University Mainz, Germany

***Corresponding author:**

Eleni Gourni

Department of Nuclear Medicine

Bern University Hospital, Switzerland

Rosenbühlgasse 25, 3010 Bern, Switzerland.

Email: eleni.gourni@insel.ch

Tel.: +41 (0)31 664 0507.

**METHODS**

**Reagents and instrumentation**

All reagents were of the best grade available and were purchased from common suppliers. All culture reagents were from Gibco BRL, Life Technologies (Grand Island, NY). The human prostate adenocarcinoma cell line PC3 (CLS-300312, Lot number 816SF) and cancer associated prostate fibroblast cell line (hTERT PF179T, ATCC CRL-3290, Lot Number 0303) were obtained from Cell Lines Service GmbH (CLS, Eppelheim, Germany) and the American Type Culture Collection (ATCC, Manassas, Virginia), respectively. The human serum was commercially available by Sigma (H4522). The GalliaPharm® Ge-68/Ga-68 generator was available from Eckert & Ziegler (Berlin, Germany). [^177^Lu]LuCl_3_ (n.c.a.) in 0.04 M HCl was obtained from DSD-Pharma GmbH (Purkersdorf near Vienna, Austria). The quality control of the precursor and the radiotracers was performed by an analytical Reverse-Phase High Performance Liquid Chromatography (RP-HPLC) on an analytical Nucleosil 120-5 column C18 (250 x 4.5 mm) applying a linear gradient of 15-90% solvent B in 30 min at a flow rate of 1 mL/min. (solvent A, 0.1% trifluoroacetic acid (TFA)/water (H_2_O); solvent B, 0.1%TFA/acetonitrile (ACN). Ultraviolet detection was performed using an Agilent detector at 214 nm. For radioactivity measurement, a Na(TI) well-type scintillation Gina star was used. The radioligand solutions for the experiments were prepared by dilution with 0.9% NaCl (Bichsel AG, Interlaken, Switzerland).

Quantitative γ-counting was performed with a COBRA II γ-system well counter from Packard Instrument (USA). For μPET/CT studies, a dedicated micro-PET/SPECT/CT scanner (Albira Si; Bruker Biospin, Ettlingen, Germany) was used.

Mice were purchased from Janvier Labs (Rue du Genest, 53940 Le Genest-Saint-Isle, France), pentobarbital natrium (150mg/kg) from Streuli Pharma SA (Uznach, Switzerland).

PC3 tumors xenografts aimed for autoradiography were snap frozen in liquid nitrogen and then cut in Leica cryostat to obtain 7 um sections.

**Radiochemistry / Quality control of the radiotracers**

**Radiolabeling with gallium-68**

[^68^Ga]Ga**-**DOTAGA.Glu_2_.(FAPI)_2_ and [^68^Ga]Ga**-**DOTAGA.PEG_2_.Glu.(FAPI)_2_ were prepared within 5 min, using the Modular-Lab PharmTracer module by Eckert&Ziegler (Berlin, Germany). The radiolabeling performance of all precursors was assessed at pH 4.6 with 1 M HEPES buffer at 90 °C for 5 min using, in each case, the minimum conjugate amount. Briefly, the Ge-68/Ga-68 generator was eluted with 5 mL 0.1 N HCl and the eluate (~300 MBq) was loaded onto a cation exchange column (Strata-XC, Phenomenex). Gallium-68 was eluted with 700 μL of a mixture of 5.5 M NaCl / 0.1 M HCl (prepared by mixing 0.53 mL of ultrapure 30% aq. HCl and 49.5 mL of 5.5 M NaCl) directly into a vial containing 2 mL 1 M HEPES (pH 5.3), 200 μL of EtOH, and 23.5 μg (approximately 15 nmol) of the conjugate, followed by SepPak C-18 purification to remove uncomplexed gallium-68. The purified [^68^Ga]Ga-DOTAGA.Glu.(FAPI)_2_ and [^68^Ga]Ga-DO3A.Glu.(FAPI)_2_ were eluted in 1.4 mL of a solution of 6.2% EtOH in PBS.

The quality control of [^68^Ga]Ga**-**DOTAGA.Glu_2_.(FAPI)_2_ and [^68^Ga]Ga**-**DOTAGA.PEG_2_.Glu.(FAPI)_2_ was performed by radio-HPLC as described in the reagents and instrumentation. The presence of free gallium-68 and ^68^Ga-labelled colloid in the [^68^Ga]Ga**-**DOTAGA.Glu_2_.(FAPI)_2_ and [^68^Ga]Ga**-**DOTAGA.PEG_2_.Glu.(FAPI)_2_ preparations was quantified by radio thin layer chromatography (Radio-TLC) using ITLC-SG-plates (Glass microfiber chromatography paper impregnated with silica gel) and 0.1 M Na-citrate buffer as mobile phase eluent.

**Radiolabeling with lutetium-177**

[^177^Lu]Lu-DOTAGA.Glu_2_.(FAPI)_2_ and [^177^Lu]Lu-DOTAGA.PEG_2_.Glu.(FAPI)_2_ were prepared by dissolving 7.8 μg (approximately 5 nmol) of DOTAGA.Glu_2_.(FAPI)_2_ and DOTAGA.PEG_2_.Glu.(FAPI)_2_ in 200-250 μL HEPES buffer (1 M, pH 5.3), followed by incubation with [^177^Lu]Lu^3+^ (30 - 200 MBq) at 90 °C for 30 min. Both products were used without any further purification step.

The quality control of [^177^Lu]Lu-DOTAGA.Glu_2_.(FAPI)_2_ and [^177^Lu]Lu-DOTAGA.PEG_2_.Glu.(FAPI)_2_ was performed by radio-HPLC and radio thin layer chromatography (radio-TLC) as described in the reagents and instrumentation.

After the labelling with gallium-68 or lutetium-177 and the quality control of the generated radiotracers, one equivalent of ^nat^LuCl_3_ x 6H_2_O were added to the relevant radiolabelling solutions. The final solutions were incubated at 90 °C for 30 min to obtain structurally characterized homogeneous ligands which were used for the saturation binding studies. The homogeneity was determined by HPLC, showing one peak.

**Lipophilicity / Protein binding studies**

The lipophilicity (LogD_octanol/PBS,_ pH 7.4) of [^68^Ga]Ga-DOTAGA.Glu_2_.(FAPI)_2_, [^68^Ga]Ga-DOTAGA.PEG_2_.Glu.(FAPI)_2_, [^177^Lu]Lu-DOTAGA.Glu_2_.(FAPI)_2_ and [^177^Lu]Lu-DOTAGA. PEG_2_.Glu.(FAPI)_2_ was estimated by the “shake-flask” method: The labelled conjugates (50 pmol; 0.3 MBq) were added to a 1:1 mixture of 1-octanol (500 μL) and PBS (500 μL, pH 7.4). The mixture was intermittently vortexed for 1 hour to reach the equilibrium and then centrifuged (3000 rpm) for 10 minutes. From each phase, an aliquot (100 μL) was pipetted out and measured in a gamma-counter. Each measurement was repeated five times. Care was taken to avoid cross-contamination between the phases. The partition coefficient was calculated as the average log ratio of the radioactivity in the organic fraction and the PBS fraction.

[^68^Ga]Ga-DOTAGA.Glu_2_.(FAPI)_2_, [^68^Ga]Ga-DOTAGA.PEG_2_.Glu.(FAPI)_2_, [^177^Lu]Lu-DOTAGA.Glu_2_.(FAPI)_2_ and [^177^Lu]Lu- DOTAGA.PEG_2_.Glu.(FAPI)_2_ (50 pmol; 0.3 MBq) were incubated with commercially available human serum (0.5 mL) at 37 °C for 30 and 60 minutes. When the incubating period was completed, proteins were precipitated with a solution of 1 mL MeOH/ACN (1:1). Centrifugation (10 minutes, 3000 rpm) was performed for the separation of proteins. After careful separation of the two phases, the respective activities were measured in a gamma-counter, followed by determination of the percentage of the radiotracer which binds to the serum proteins.

**Cell lines**

The human prostate adenocarcinoma cell line PC3 (RRID:CVCL 0035) and the prostate-derived cancer-associated fibroblasts CAF cells (hTERT PF179T, ATCC) have been used in the present study. PC3 cell lines were cultured in Dulbecco's Modified Eagle Medium (DMEM) with low glucose (1g/L) and F-12 Nutrient Mix with GlutaMAX™-I in a 1:1 mixture ratio. The CAFs were cultured using the Eagle's Minimal Essential Medium (EMEM) supplemented with sodium bicarbonate 1500 mg/L and puromycin 1 μg/mL. The cultivation media were supplemented with 5% fetal bovine serum for PC3 cells and 10% for CAF cells, along with penicillin (100 U/mL) and streptomycin (100 µg/mL). Both cell lines were cultured at 37°C and 5% CO_2_.

**Saturation binding studies**

For receptor saturation analysis, the CAF cell line overexpressing FAP were seeded at a density of 0.8-1 million cells per well in 6-well plates, pre-coated with BME (Cultrex Reduced Growth Factor Basement Membrane Extract, RGF BME, R&D System, Minneapolis, USA) to enhance cell attachment. The cells were incubated overnight with medium (EMEM supplemented and containing 10% FBS, 100 U/mL penicillin and 100 μg/mL streptomycin). The next day, the medium was removed and the cells were incubated for 30 minutes at 37 °C, with 0.8 mL of fresh medium. Afterwards, the plates were placed on ice for 30 minutes followed by incubation with increasing concentrations of ^177/nat^Lu-labelled DOTAGA.Glu_2_.(FAPI)_2_ and DOTAGA.PEG_2_.Glu.(FAPI)_2_ (0.1-10 nM final concentration in the wells). (To ensure that no free FAPI ligand remained after the radiolabeling step, we performed in-site complexation using 3 equivalents of ^nat^Lu under identical conditions used for the radiolabeling with ^177^Lu). After the addition of the radioligands, the cells were incubated for 120 minutes at 4 °C. Non-specific binding was determined in the presence of UAMC1110 at a final concentration of 1 μM. After the completion of the incubation, the cells were washed twice with ice-cold PBS, followed by solubilization with 1 M NaOH. The cell-associated radioactivity was measured using a gamma-counter. Specific binding was plotted against the total molar concentration of the added radiotracer. The K_d_ values and the concentration of the radiotracer required to saturate the receptors (B_max_) were determined by nonlinear regression using GraphPad (Prism 10 Graph Pad Software, San Diego, CA). For all the cell studies the values are normalized for 1x10^6^ cells per well and all data are from two independent experiments with triplicates in each experiment.

**Internalization studies**

For internalization experiments, CAF cells were seeded into 6-well plates pre-coated with BME and treated as described at the saturation binding studies. On the day of the experiment, approximately 2.5 pmol (100 µL) of the radiotracer was added to the medium (total volume 1.5 mL) and the cells were incubated (in triplicates) for 0.5, 1, 2, 4 and 6 hours at 37 °C, 5% CO_2_ for the ^177^Lu-labeled tracers. To determine nonspecific membrane binding and internalization, excess of UAMC1110 (final concentration 1 μΜ) was added to selected wells. At each time point, the internalization was stopped by putting the plates on ice, removing the medium and washing the cells twice with ice-cold PBS. To remove the receptor-bound radioligand, an acid wash was carried out twice with a 0.1 M glycine buffer pH 2.8 for 5 minutes on ice. Finally, cells were solubilized with 1 M NaOH. The radioactivity of the culture medium, the receptor-bound, and the internalized fractions were measured in a gamma-counter.

**Externalization studies**

The externalization rate of [^177^Lu]Lu-DOTAGA.Glu_2_.(FAPI)_2_ and [^177^Lu]Lu- DOTAGA.PEG_2_.Glu.(FAPI)_2_ was studied after CAFs were incubated for 2 hours with 2.5 pmol/well of each ^177^Lu-lebeled radioligand. After the completion of the 2 hours of incubation, the medium was removed and CAF cells were washed with 1 mL of cold PBS (x2). The cells were then exposed to an acid wash with glycine buffer, as described above, to dissociate cell surface-bound radioligand, and the fractions were collected. Fresh medium (1 mL/well) was added to the cells and were incubated at 37 °C. At different time points (10, 20, 30, 60, 120, 240 and 1440 minutes) the external medium was removed (followed by two washing steps with cold PBS). Finally, cells were solubilized with 1 M NaOH. The radioactivity of the culture medium, the receptor-bound, the internalized and the externalized fractions were measured in a gamma-counter. The externalized fraction of radioactivity was expressed as the percentage of the total internalized amount.

**Biodistribution studies**

To demonstrate the specificity of binding, mice were co-injected with 600 pmol of each Ga-68 radiotracer along with 20 nmol of cold ligand (total injected volume: 100 µL) and biodistribution studies were performed 1 hour after the injection.

Blocking studies were performed by co-injection of 600 pmol of each Lu-177 radiotracer along with 20 nmol of the respective non-labeled ligand (total injected volume 100 µL). Biodistribution studies were performed 4 hours after injection.

All mice were terminally anesthetized with an overdose of pentobarbital natrium (150 mg/kg; Streuli Pharma SA) administered via intraperitoneal injection. Selected organs were then dissected and weighted. The radioactivity in tissue samples was measured using a gamma-counter. Biodistribution data are presented as percent of injected activity per gram of tissue (% I.A./g) and are means ± SD (n = 3).

**Small-Animal PET / SPECT/CT imaging**

PET images were corrected for gallium-68 decay and reconstructed with 1 iteration of Ordered Subset Expectation Maximization (OSEM) algorithm using a voxel size of 0.75 mm. Partial Volume correction along with a Point Spread Function iterative deconvolution were applied. The images were normalized and generated using PMOD software. The color scale of the PET images was set from 0 to 12 % I.A./mL allowing for qualitative comparison among the images. The CT was carried out using step and shoot mode and employed 45 kVp and 400 μA as settings. The CT data were reconstructed using Filtered Back Projection (FBP) algorithm and a voxel size of 0.125 mm.  The color scale of the CT is ranging from 150 to 700 Hounsfield Units (HUs).

SPECT images were acquired using a 208 keV ± 20 % energy window and a Cerrobase (Bi-Pb alloy) 30 mm thick collimator, with a pinhole 22 mm thick tungsten lens, 60 projections and 180 – 1440 s per projection. SPECT data were reconstructed using the Ordered Subset Expectation Maximization (OSEM) algorithm with 2 iterations of 5 subsets and a voxel size of 0.5 mm. The reconstructed data were corrected for lutetium-177 decay, normalized and filtered, using a Gaussian 3D algorithm with a 1.5 mm isotropic kernel, and generated using PMOD software. The color scale of the SPECT images was set from 0 to 16 % I.A./mL, allowing for qualitative comparison among the images. The CT was carried out using step-and-shoot mode, employing 45 kVp and 400 μA as settings. CT data were reconstructed using FBP algorithm and a voxel size of 0.125 mm. The color scale of the CT is ranging from 150 to 700 HUs.

**Blood and organs clearance kinetics**

Blood samples were weighed, and the radioactivity was counted in the γ-counter. The data were calculated as the counts per gram of blood (Counts/g) and plotted versus time. The initial and terminal half-lives of each radioligand in blood were determined using GraphPad Prism 10 software, employing the non-linear regression Two-Phase Decay Model. The biological half-lives of the radioligands in various organs were calculated accordingly, using the biodistribution data and plotting %I.A./g against time. The non-linear regression One-Phase Decay Model was applied.

**Metabolic stability in murine plasma**

Healthy mice (n = 2) were injected with 600 pmol (~7 MBq) of [^177^Lu]Lu-DOTAGA.Glu_2_.(FAPI)_2_ and [^177^Lu]Lu-DOTAGA.PEG_2_.Glu.(FAPI)_2_ (in a total injected volume of 100 µL of 0.9% NaCl) and sacrificed 15 min post-injection (p.i.). Blood was immediately transferred to pre-chilled heparinized tubes and centrifuged (5 min, 1700 g, 4 °C) for plasma isolation. The isolated plasma was then transferred into a 2 mL Eppendorf and a double volume of a 1:1 (v/v) ACN:MeOH solution was added to induce the precipitation of the plasma proteins. The proteins were separated by centrifugation (10 min, 9660 g, 4 °C) and the two phases (precipitated proteins and supernatant) were carefully separated.

To evaluate the in vivo metabolic stability of [^177^Lu]Lu-DOTAGA.Glu_2_.(FAPI)_2_ and [^177^Lu]Lu-DOTAGA.PEG_2_.Glu.(FAPI)_2_ in blood circulation and determine if and to which extent the remaining circulating activity in blood is subjected to enzymatic degradation, samples from the supernatant were analyzed by radio-HPLC (from tested time point 15 min p.i.).

**In vivo monotherapy and combination therapy of PC3 tumor bearing mice using [^177^Lu]Lu-DOTAGA.Glu_2_.(FAPI)_2_**

**FIGURE S1:** Schematic representation of the monotherapy and combination therapy studies using [^177^Lu]Lu-DOTAGA.Glu_2_.(FAPI)_2_ in PC3 mice.

**RESULTS**

**Quality control of the radiotracers / Stability**

Based on the amount of the precursor which was used for the radiolabeling and assuming we lose about 20% during the labeling, the apparent molar activities (A_m_) were in the range of 6 to 30 GBq/µmol (not decay corrected).

With regard to the detection of the formation of colloids by ITLC, using radio-TLC eluent (0.1 M Na-citrate), [^68^Ga]Ga-DOTAGA.PEG_2_.Glu.(FAPI)_2_, [^68^Ga]Ga-DOTAGA.Glu_2_.(FAPI)_2_ and ^68^Ga-labelled colloid remain immobilized at the starting point, whereas free gallium-68 moves with the mobile phase. The same systems have been applied for the ^177^Lu-labelled tracers.

**Figure S2.** Representative radio-HPLC chromatograms of [^68^Ga]Ga-DOTAGA.PEG_2_.Glu.(FAPI)_2_, [^68^Ga]Ga-DOTAGA.Glu_2_.(FAPI)_2_, [^177^Lu]Lu-DOTAGA.PEG_2_.Glu.(FAPI)_2_ and [^177^Lu]LuDOTAGA.Glu_2_.(FAPI)_2_

**Figure S3**. Radio-TLC profiles of [^68^Ga]Ga-DOTAGA.PEG_2_.Glu.(FAPI)_2_, [^68^Ga]Ga-DOTAGA.Glu_2_.(FAPI)_2_, [^177^Lu]Lu-DOTAGA.PEG_2_.Glu.(FAPI)_2_ and [^177^Lu]LuDOTAGA.Glu_2_.(FAPI)_2_ (R_f_ = 0) in 0.1 M sodium citrate buffer (mobile phase), indicating the radiochemical purity (RCP) after radiolabeling (10 min) expressed as percentage of intact tracer detected over total radioactivity.

**Figure S4**. Radio-TLC profiles of [^177^Lu]Lu-DOTAGA.PEG_2_.Glu.(FAPI)_2_ (R_f_ = 0) in 0.1 M sodium citrate buffer (mobile phase), indicating the radiochemical purity (RCP) at several time points after radiolabeling (1, 24, 48 and 168 h) expressed as percentage of intact tracer detected over total radioactivity.

**Figure S5**. Radio-TLC profiles of [^177^Lu]LuDOTAGA.Glu_2_.(FAPI)_2_ (R_f_ = 0) in 0.1 M sodium citrate buffer (mobile phase), indicating the radiochemical purity (RCP) at several time points after radiolabeling (1, 24, 48 and 168 h) expressed as percentage of intact tracer detected over total radioactivity.

| **[^68^Ga]Ga-DOTAGA.Glu_2_.(FAPI)_2_** | | | | | **[^68^Ga]Ga-DOTAGA.PEG_2_.Glu.(FAPI)_2_** | | | |
| --- | --- | --- | --- | --- | --- | --- | --- | --- |
| **Organs** | **1h** | **2h** | **3h** | **Blocking 1h** | **1h** | **2h** | **3h** | **Blocking 1h** |
| **Blood**  **Heart**  **Gallbladder**  **Liver**  **Spleen**  **Lung**  **Kidney**  **Stomach**  **Intestine**  **Adrenal**  **Pancreas**  **Muscle**  **Bone**  **Tumor**  **Salivary glands** | 3.3±0.1  1.3±0.2  1.5±0.6  3.9±0.2  5.7±1.3  2.7±1.6  4.7±0.1  1.0±0.1  1.4±0.1  3.1±2.1  1.7±0.1  1.4±0.2  5.8±0.9  13.0±3.1  5.4±0.1 | 2.9±0.1  1.1±0.1  1.5±0.4  5.1±0.9  5.9±0.7  2.5±0.3  4.2±0.5  0.9±0.2  1.2±0.3  2.2±1.1  1.7±0.3  1.3±0.2  5.6±0.6  13.6±1.4  4.5±1.1 | 2.8±0.2  1.1±0.1  0.7±0.4  5.4±0.6  6.3±0.4  2.4±0.4  4.5±0.6  0.9±0.1  1.0±0.2  2.0±0.6  1.8±0.4  1.3±0.3  6.3±0.4  13.9±1.9  4.4±0.9 | 1.5±0.1  0.5±0.1  3.2±0.5  3.0±0.03  2.7±0.2  1.8±0.02  4.3±0.1  0.4±0.01  0.6±0.1  2.9±1.6  0.4±0.0  0.3±0.03  0.9±0.1  1.2±0.1  0.7±0.02 | 2.9±0.2  1.3±0.1  5.2±1.0  2.4±0.2  1.4±0.1  3.3±0.2  6.2±0.4  2.6±2.8  1.3±0.2  6.6±0.6  2.2±0.02  1.7±0.1  6.8±0.6  19.2±2.8  5.7±0.4 | 2.7±0.3  1.0±0.1  3.2±3.0  1.5±0.04  1.1±0.1  1.7±0.03  3.3±0.3  0.8±0.2  1.1±0.1  1.5±0.8  1.7±0.3  1.5±0.4  4.6±1.1  18.3±3.3  5.5±1.0 | 2.6±0.3  1.0±0.2  1.3±0.8  2.1±0.9  1.2±0.1  1.7±0.01  3.9±0.6  0.8±0.2  1.2±0.1  1.4±1.9  1.9±0.2  1.5±0.4  5.4±0.8  21.4±5.6  5.6±0.7 | 1.7±0.1  0.7±0.03  2.7±0.4  2.3±0.1  1.2±0.02  3.3±0.3  8.3±0.3  0.7±0.02  1.1±0.1  4.1±0.4  0.8±0.2  0.2±0.2  1.1±0.04  1.5±0.1  0.9±0.01 |

**Biodistribution Studies**

**Table S1**. Biodistribution data of [^68^Ga]Ga-DOTAGA.Glu_2_.(FAPI)_2_ and [^68^Ga]Ga-DOTAGA.PEG_2_.Glu.(FAPI)_2_ in PC3-mice at 1, 2, and 3 h p.i along with blocking studies data

**Table S2**. Tumor to organ (blood, liver, kidney, bone, salivary glands) ratios of [^68^Ga]Ga-DOTAGA.Glu_2_.(FAPI)_2_ and [^68^Ga]Ga-DOTAGA.PEG_2_.Glu.(FAPI)_2_ in PC3-mice at 1, 2 and 3 h p.i.

|  | **[^68^Ga]Ga-DOTAGA.Glu_2_.(FAPI)_2_** | | | **[^68^Ga]Ga-DOTAGA.PEG_2_.Glu.(FAPI)_2_** | | |
| --- | --- | --- | --- | --- | --- | --- |
|  | **1h** | **2h** | **3h** | **1h** | **2h** | **3h** |
| **Tumor/Blood** | 3.9±0.9 | 4.7±0.6 | 5.1±0.96 | 6.6±0.9 | 6.8±1.6 | 8.5±3.03 |
| **Tumor/Liver** | 3.3±0.7 | 2.8±0.7 | 2.6±0.4 | 8.0±1.7 | 12.5±2.3 | 11.3±3.5 |
| **Tumor/Kidney** | 2.8±0.6 | 3.3±0.7 | 3.1±0.6 | 3.1±0.7 | 5.6±0.7 | 5.5±0.7 |
| **Tumor/Bone** | 2.3±0.7 | 2.5±0.5 | 2.2±0.2 | 2.8±0.3 | 4.2±1.8 | 4.1±1.4 |
| **Tumor/Salivary glands** | 2.7±0.6 | 3.1±0.5 | 3.2±0.5 | 3.3±0.4 | 3.5±1.3 | 3.9±1.4 |

**Table S3**. Biodistribution data and tumor-to-organ ratios of [^177^Lu]Lu-DOTAGA.Glu_2_.(FAPI)_2_ in PC3-mice at 4, 24, 48, 72, 96 and 168 h p.i. along with blocking studies data at 4 h p.i. Data have been calculated as %I.A./g of tissue and are presented as mean ± SD (n = 3).

| **[^177^Lu]Lu-DOTAGA.Glu_2_.(FAPI)_2_** | | | | | | | | |
| --- | --- | --- | --- | --- | --- | --- | --- | --- |
| **Organs** | **4h** | **24h** | **48h** | **72h** | **96h** | **168h** | **Blocking 4h** |  |
| **Blood**  **Heart**  **Gallbladder**  **Liver**  **Spleen**  **Lung**  **Kidney**  **Stomach**  **Intestine**  **Adrenal**  **Pancreas**  **Muscle**  **Bone**  **Tumor**  **Salivary glands** | 2.6±0.3  1.3±0.1  0.7±0.3  1.6±0.2  1.03±0.1  1.6±0.2  3.1±0.5  1.0±0.1  1.03±0.1  3.6±0.4  2.6±0.4  2.3±0.2  4.6±0.3  15.2±3.2  9.7±1.7 | 1.3±0.1  1.0±0.04  0.5±0.1  3.0±0.4  1.5±0.3  0.9±0.1  2.8±0.6  0.7±0.04  0.7±0.1  2.8±0.01  1.6±0.2  1.7±0.3  2.9±0.6  10.6±1.9  7.0±1.0 | 0.5±0.02  0.8±0.1  0.3±0.1  3.2±0.1  1.5±0.03  0.5±0.03  2.3±0.2  0.5±0.1  0.4±0.04  2.1±0.3  1.0±0.1  1.0±0.2  1.9±0.2  7.3±0.7  4.4±0.3 | 0.2±0.01  0.6±0.03  0.1±0.04  2.6±0.2  1.1±0.02  0.5±0.3  1.5±0.2  0.3±0.02  0.3±0.02  1.8±0.3  0.6±0.03  0.7±0.1  1.4±0.3  4.4±0.08  2.5±0.1 | 0.1±0.0  0.5±0.02  0.1±0.1  2.2±0.1  1.1±0.2  0.3±0.1  1.2±0.2  0.2±0.04  1.6±2.4  1.2±0.1  0.5±0.1  0.4±0.1  1.4±0.3  4.2±0.5  2.4±0.1 | 0.01±0.0  0.2±0.02  0.02±0.0  1.3±0.04  0.6±0.1  0.1±0.02  0.5±0.1  0.1±0.02  0.6±0.9  0.7±0.1  0.2±0.01  0.2±0.1  0.8±0.3  2.0±0.4  0.9±0.2 | 0.2±0.02  0.2±0.01  0.0±0.0  1.3±0.01  0.5±0.03  1.0±0.2  3.8±0.5  0.2±0.01  0.5±0.1  0.3±0.1  0.2±0.02  0.1±0.01  0.3±0.1  0.9±0.1  0.4±0.03 |  |
|  |  |  |  |  |  |  |  |  |
| **Tumor/Blood**  **Tumor/Liver**  **Tumor/Kidney**  **Tumor/Bone**  **Tumor/Salivary glands** | 5.9±2.0  9.9±3.2  4.9±0.4  3.3±0.6  1.6±0.5 | 8.1±1.0  3.5±0.2  3.9±0.7  3.7±0.9  1.6±0.4 | 14.0±1.7  2.3±0.2  3.2±0.5  3.8±0.3  1.7±0.1 | 22.2±1.2  1.7±0.1  3.0±0.3  3.4±0.7  1.8±0.1 | 44.6±3.8  1.9±0.2  3.5±0.3  3.2±0.9  1.8±0.2 | 130.6±15.9  1.5±0.2  3.9±0.3  2.5±0.5  2.1±0.2 |  |  |

**Table S4**. Biodistribution data and tumor to organ ratios of [^177^Lu]Lu-DOTAGA.PEG_2_.Glu.(FAPI)_2_ in PC3-mice at 4, 24, 48, 72, 96 and 168 h p.i. along with blocking studies data at 4 h p.i. Data have been calculated as %I.A./g of tissue and are presented as mean ± SD (n = 3).

|  | **[^177^Lu]Lu-DOTAGA.PEG_2_.Glu.(FAPI)_2_** | | | | | | | |
| --- | --- | --- | --- | --- | --- | --- | --- | --- |
|  | | **4h** | **24h** | **48h** | **72h** | **96h** | **168h** | **Blocking 4h** |
| **Blood**  **Heart**  **Gallbladder**  **Liver**  **Spleen**  **Lung**  **Kidney**  **Stomach**  **Intestine**  **Adrenal**  **Pancreas**  **Muscle**  **Bone**  **Tumor**  **Salivary glands** | | 2.0±0.4  0.9±0.1  2.5±1.4  2.0±0.3  1.1±0.3  1.2±0.2  3.2±0.8  0.7±0.2  0.9±0.3  2.7±0.9  1.7±0.2  1.6±0.5  5.4±1.0  15.7±2.3  5.8±0.8 | 0.7±0.1  0.4±0.04  0.7±0.4  1.6±0.1  0.6±0.1  0.5±0.1  1.3±0.1  0.3±0.04  0.4±0.1  1.4±0.2  0.8±0.04  0.8±0.1  1.8±0.3  6.4±0.8  2.6±0.4 | 0.3±0.04  0.2±0.01  0.3±0.1  1.3±0.1  0.4±0.1  0.2±0.03  0.6±0.1  0.1±0.01  0.2±0.02  0.9±0.2  0.4±0.03  0.3±0.04  0.9±0.2  3.7±0.3  1.0±0.1 | 0.1±0.01  0.1±0.01  0.2±0.1  0.9±0.03  0.3±0.04  0.4±0.5  0.4±0.02  0.1±0.01  0.1±0.02  0.5±0.1  0.1±0.02  0.1±0.01  0.5±0.1  1.9±0.3  0.4±0.03 | 0.02±0.0  0.03±0.02  0.2±0.2  0.8±0.1  0.3±0.1  0.04±0.01  0.2±0.03  0.02±0.0  0.04±0.0  0.2±0.1  0.1±0.0  0.04±0.01  0.3±0.1  1.2±0.02  0.2±0.02 | 0.0±0.0  0.01±0.0  0.02±0.0  0.5±0.1  0.2±0.1  0.02±0.01  0.1±0.01  0.01±0.0  0.01±0.0  0.1±0.02  0.01±0.0  0.0±0.0  0.1±0.02  0.4±0.2  0.03±0.01 | 0.2±0.02  0.2±0.01  0.0±0.0  1.3±0.01  0.5±0.03  1.0±0.2  3.8±0.5  0.2±0.01  0.5±0.1  0.3±0.1  0.2±0.02  0.1±0.01  0.3±0.1  0.9±0.1  0.4±0.03 |
|  | |  |  |  |  |  |  |  |
| **Tumor/Blood**  **Tumor/Liver**  **Tumor/Kidney**  **Tumor/Bone**  **Tumor/Salivary glands** | | 7.6±2.1  7.7±2.0  5.0±1.8  2.8±0.6  2.6±0.7 | 8.9±0.4  4.0±0.6  4.9±0.9  3.6±0.4  2.5±0.2 | 14.3±2.0  2.9±0.2  6.2±0.4  4.4±0.8  3.6±0.5 | 30.7±7.5  2.1±0.4  4.8±0.9  4.2±1.1  4.8±0.5 | 68.7±16.8  1.6±0.3  5.6±0.8  4.5±0.8  6.7±0.7 | 199.2±37.9  0.9±0.2  5.8±1.3  4.0±0.7  8.9±2.0 |  |

**Organ clearance kinetics**

**Table S5**: Organ clearance in terms of biological half-life for [^177^Lu]Lu-DOTAGA.PEG_2_.Glu.(FAPI)_2_ and [^177^Lu]Lu-DOTAGA.Glu_2_.(FAPI)_2_

| **Organ** | **Half-life (h) for [^177^Lu]Lu-DOTAGA.PEG_2_.Glu.(FAPI)_2_** | **Half-life (h) for [^177^Lu]Lu-DOTAGA.Glu_2_.(FAPI)_2_** |
| --- | --- | --- |
| **Liver** | 70.58 | 324.40 |
| **Kidney** | 18.42 | 67.14 |
| **Pancreas** | 19.58 | 34.24 |
| **Bone** | 14.01 | 45.41 |
| **Salivary glands** | 18.19 | 40.24 |
| **Tumor** | 19.04 | 44.48 |

**Table S6:** Comparison of different ^177^Lu-labelled FAP derivatives (peptides, albumin binder conjugates dimers and tetramers) in terms of tumor and organ uptake at selected timepoints post injection

| Compound | Tumor  (%IA/g) | Liver  (%IA/g) | Kidney (%IA/g) | Tumor model | Ref. |
| --- | --- | --- | --- | --- | --- |
| FAP-HXN  peptide | 16.68±0.82  4 h p.i.  12.49±3.00 48h p.i. | 1.38 ±0.07 4 h p.i. | 4.43±0.59  4 h p.i. | HEK-293-FAP tumor xenografts | [1] |
| FAP-2286  peptide | 3.42 ± 1.06  4 h p.i.  1.67 ± 0.50 24h p.i. | 0.11 ± 0.02 4 h p.i. | 5.15 ± 0.65  4 h p.i. | HT-1080 xenograft | [2] |
| FAP-2286  peptide | 22.99 ± 3.13  4 h p.i.  13.49 ± 1.95 24 h p.i. | 0.11 ± 0.01 4 h p.i. | 3.98 ± 0.42  4 h p.i. | HEK-293 xenografts | [2] |
| FAPI-46-Ibu | 8.40 ± 1.97  4 h p.i.  5.09 ± 0.64  24 h p.i. | 0.96 ± 0.08, 4 h p.i. | 1.80 ± 0.15 4 h p.i. | HT-1080 xenograft | [2] |
| FAPI-46-Ibu | 5.03 ± 2.84  4 h p.i.  3.14 ± 0.49  24 h p.i. | 0.75 ± 0.11 4 h p.i. | 1.33 ± 0.17  4 h p.i. | HEK-293 xenografts | [2] |
| FAPI-46-EB | 5.02 ± 0.29  4 h p.i.  5.44 ± 1.03  24 h p.i. | 2.80 ± 0.26 4 h p.i. | 4.33 ± 0.12 4 h p.i. | HT-1080 xenograft | [2] |
| FAPI-46-EB | 12.58 ± 2.83  4 h p.i.  16.69 ± 1.01 24 h p.i. | 3.06 ± 0.36 4 h p.i. | 4.70 ± 1.03  4 h p.i. | HEK-293 xenografts | [2] |
| TEFAPI-06  Albumin binder | 8.68 ± 0.73  24 h p.i. | 2.98 ± 0.64 24 h p.i. | 3.20 ± 0.84  24 h p.i. | pancreatic cancer PDX– xenografts | [3] |
| TEFAPI-07  Albumin binder | 7.87 ± 2.08  24 h p.i. | 3.21 ± 0.81, 24 h p.i. | 8.67 ± 2.30  24 h p.i. | pancreatic cancer PDX– xenografts | [3] |
| DOTA-4P(FAPI)_4_ | 21.4 ± 1.7  24 h p.i.  19.2 ± 0.6  48 h p.i. | 6.3 ± 0.5  48 h p.i. | 6.6 ± 0.2  48 h p.i. | HT-1080-FAP tumor xenografts | [4] |
| DOTAGA.Glu_2_.(FAPI)_2_ | 15.2 ± 3.2  4 h p.i.  10.6 ± 1.9  24 h p.i. | 3.0 ± 0.4, 24 h p.i. | 2.8 ± 0.6  24 h p.i. | PC3 xenografts | Present study |
| DOTAGA.PEG_2_.Glu.(FAPI)_2_ | 15.7 ± 2.3  4 h p.i.  6.4 ± 0.8  24 h p.i. | 0.5 ± 0.1, 24 h p.i. | 1.3 ± 0.1  24 h p.i. | PC3 xenografts | Present study |

**Table S7**: Comparison of FAPI monomers, 1^st^ generation, 2^nd^ generation and 3^rd^ generation FAPI homodimers

| **Compound** | **LogD_octanol/PBS_** | **%**  **Protein binding**  **(30 min)** | **% Protein binding (60 min)** | **K_d_**  **(nM)** | **B_max_ (nM)** | **Ref.** |
| --- | --- | --- | --- | --- | --- | --- |
| **FAPI monomers** | | | | | |  |
| [^68^Ga]Ga-DOTA.SA.FAPI | −3.38 ± 0.03 | 10.6 ± 3.9 |  | 0.8 ± 0.22 | 1.6 ± 0.53 | [5] |
| [^177^Lu]Lu-DOTA.SA.FAPI | −2.86 ± 0.06 | 9.9 ± 4.4 |  | 0.4 ± 0.03 | 0.7 ± 0.08 | [5] |
| **1^st^ Generation FAPI homodimers** | | | | | |  |
| [^68^Ga]Ga-DOTAGA.(SA.FAPI)_2_ | −1.83 ± 0.02 | 18 ± 1.1 |  | 1.2 ± 0.26 | 0.5 ± 0.03 | [5] |
| [^177^Lu]Lu-DOTAGA.(SA.FAPI)_2_ | −1.71 ± 0.03 | 25.3 ± 0.8 |  | 1.35 ± 0.7 | 0.4 ± 0.05 | [5] |
| **2^nd^ Generation FAPI homodimers** | | | | | |  |
| [^68^Ga]Ga-DOTAGA.Glu.(FAPI)_2_ | −2.90 ± 0.10 | 9.4 ± 2.2 | 6.3 ± 0.4 | 0.8 ± 0.2 | 0.7 ± 0.04 | [6] |
| [^177^Lu]Lu-DOTAGA.Glu.(FAPI)_2_ | −3.00 ± 0.10 | 9.2 ± 0.03 | 7.4 ± 0.5 | 0.7 ± 0.1 | 0.4 ± 0.02 | [6] |
| **3^rd^ Generation FAPI homodimers** | | | | | |  |
| [^68^Ga]Ga-DOTAGA.(Glu)_2_.(FAPI)_2_ | −3.49 ± 0.25 | 11.3 ± 0.4 | 8.7 ± 0.4 |  |  | Present study |
| [^68^Ga]Ga-DOTAGA.PEG_2_.(FAPI)_2_ | −3.04 ± 0.28 | 8.4 ± 0.6 | 6.5 ± 0.3 |  |  | Present study |
| [^177^Lu]Lu-DOTAGA.(Glu)_2_.(FAPI)_2_ | −3.6 ± 0.3 | 6.2 ± 0.9 | 6.1 ± 0.4 | 1.5 ± 0.2 | 0.9 ± 0.05 | Present study |
| [^177^Lu]Lu-DOTAGA.PEG_2_.(FAPI)_2_ | −3.3 ± 0.1 | 4.2 ± 0.7 | 4.4 ± 0.3 | 0.9 ± 0.2 | 1.0 ± 0.07 | Present study |

**Table S8**: Preclinical combination therapy setup with FAPI, PD and mTOR [7-9]

| FAPI | Combination compound | Tumor model | Results: Tumor growth and survival | Ref. |
| --- | --- | --- | --- | --- |
| FAP-2287 | anti-PD-1 | MCA205-mFAP tumor | Tumor volume (day 10) was 266, 373,145 mm^3^ and Median survival time were 22, 16, 27 days for ^177^Lu-FAP-2287, anti-PD-1 and combination group respectively. | [8] |
| DOTA-2P(FAPI)_2_ | αPD-L1 mAb | CT26-FAP tumor | Mean survial for αPD-L1mAb was about 26 days with slow tumor growth and about about 60% survival with^177^Lu-DOTA-2P(FAPI)_2_.Combination therapy group demonstrated the best antitumor effect, with all mice achieving complete remission (CR) by day 16 post-treatment. | [7] |
| LNC1004 | anti-PD-L1 | MC38/NIH3T3-FAP mixed tumor | Median survival for αPD-L1 and ^177^Lu-LNC1004 was 25-35 days with slower tumor growth rates. combination therapy exhibited the most remarkable antitumor efficacy, with 100% survival rate. | [9] |
| DOTAGA-Glu_2_.(FAPI)_2_ | Everolimus | PC3 tumor | Mean survial for everolimus was about 34 day with slow tumor growth and about about 50% survival with^177^Lu- DOTAGA-Glu_2_.(FAPI)_2_. Combination therapy was most effective, maintaining tumor volumes<100 mm^3^ for 70 days, and 100% survival. | Present study |

**References**

1.	Rang Wang MH, Weichen Wang, Mufeng Li, Yingwei Wang and Rong Tian. Preclinical Evaluation of 68Ga/177Lu-Labeled FAP-Targeted Peptide for Tumor Radiopharmaceutical Imaging and Therapy. Journal of Nuclear Medicine. 2025;66:250-6. doi:<https://doi.org/10.2967/jnumed.124.268689>.

2.	Millul J, Koepke L, Haridas GR, Sparrer KMJ, Mansi R, Fani M. Head-to-head comparison of different classes of FAP radioligands designed to increase tumor residence time: monomer, dimer, albumin binders, and small molecules vs peptides. Eur J Nucl Med Mol Imaging. 2023;50:3050-61. doi:10.1007/s00259-023-06272-7.

3.	Xu M, Zhang P, Ding J, Chen J, Huo L, Liu Z. Albumin Binder-Conjugated Fibroblast Activation Protein Inhibitor Radiopharmaceuticals for Cancer Therapy. J Nucl Med. 2022;63:952-8. doi:10.2967/jnumed.121.262533.

4.	Pang Y, Zhao L, Fang J, Chen J, Meng L, Sun L, et al. Development of FAPI Tetramers to Improve Tumor Uptake and Efficacy of FAPI Radioligand Therapy. J Nucl Med. 2023;64:1449-55. doi:10.2967/jnumed.123.265599.

5.	Läppchen T, Bilinska A, Pilatis E, Menéndez E, Imlimthan S, Moon ES, et al. Tailoring Fibroblast-Activation Protein Targeting for Theranostics: A Comparative Preclinical Evaluation of the (68)Ga- and (177)Lu-Labeled Monomeric and Dimeric Fibroblast-Activation Protein Inhibitors DOTA.SA.FAPi and DOTAGA.(SA.FAPi)(2). Molecules. 2024;29. doi:10.3390/molecules29133093.

6.	Bilinska A, Kumar N, Gnesin S, Läppchen T, Menéndez E, Martin M, et al. Preclinical evaluation of DOTAGA.Glu.(FAPI)(2) and DO3A.Glu.(FAPI)(2) as theranostics with human dosimetry extrapolation to lutetium-177 and terbium-161 analogs. Eur J Nucl Med Mol Imaging. 2026;53:1887-901. doi:10.1007/s00259-025-07565-9.

7.	Chen J, Zhou Y, Pang Y, Fu K, Luo Q, Sun L, et al. FAP-targeted radioligand therapy with (68)Ga/(177)Lu-DOTA-2P(FAPI)(2) enhance immunogenicity and synergize with PD-L1 inhibitors for improved antitumor efficacy. J Immunother Cancer. 2025;13. doi:10.1136/jitc-2024-010212.

8.	Zboralski D, Osterkamp F, Christensen E, Bredenbeck A, Schumann A, Hoehne A, et al. Fibroblast activation protein targeted radiotherapy induces an immunogenic tumor microenvironment and enhances the efficacy of PD-1 immune checkpoint inhibition. Eur J Nucl Med Mol Imaging. 2023;50:2621-35. doi:10.1007/s00259-023-06211-6.

9.	Zhao L, Pang Y, Zhou Y, Chen J, Fu H, Guo W, et al. Antitumor efficacy and potential mechanism of FAP-targeted radioligand therapy combined with immune checkpoint blockade. Signal Transduct Target Ther. 2024;9:142. doi:10.1038/s41392-024-01853-w.
